# SVEngine: an efficient and versatile simulator of genome structural variations with features of cancer clonal evolution

**DOI:** 10.1093/gigascience/giy081

**Published:** 2018-07-05

**Authors:** Li Charlie Xia, Dongmei Ai, Hojoon Lee, Noemi Andor, Chao Li, Nancy R Zhang, Hanlee P Ji

**Affiliations:** 1 Division of Oncology, Department of Medicine, Stanford University School of Medicine, 269 Campus Drive, Stanford, CA 94305; 2 Department of Statistics, the Wharton School, University of Pennsylvania, 3730 Walnut Street, Philadelphia, PA 18014; 3 School of Mathematics and Physics, University of Science and Technology Beijing, 30 Xueyuan Road, Haidian District, Beijing 100083 P. R. China; 4 Stanford Genome Technology Center, Stanford University, 3165 Porter Drive, Palo Alto, CA 94304

**Keywords:** structural variation, next-generation sequencing, sequence analysis, locus-specific allele frequency, somatic haplotypes, cancer clonal evolution

## Abstract

**Background:**

Simulating genome sequence data with variant features facilitates the development and benchmarking of structural variant analysis programs. However, there are only a few data simulators that provide structural variants *in silico* and even fewer that provide variants with different allelic fraction and haplotypes.

**Findings:**

We developed SVEngine, an open-source tool to address this need. SVEngine simulates next-generation sequencing data with embedded structural variations. As input, SVEngine takes template haploid sequences (FASTA) and an external variant file, a variant distribution file, and/or a clonal phylogeny tree file (NEWICK) as input. Subsequently, it simulates and outputs sequence contigs (FASTAs), sequence reads (FASTQs), and/or post-alignment files (BAMs). All of the files contain the desired variants, along with BED files containing the ground truth. SVEngine's flexible design process enables one to specify size, position, and allelic fraction for deletions, insertions, duplications, inversions, and translocations. Finally, SVEngine simulates sequence data that replicate the characteristics of a sequencing library with mixed sizes of DNA insert molecules. To improve the compute speed, SVEngine is highly parallelized to reduce the simulation time.

**Conclusions:**

We demonstrated the versatile features of SVEngine and its improved runtime comparisons with other available simulators. SVEngine's features include the simulation of locus-specific variant frequency designed to mimic the phylogeny of cancer clonal evolution. We validated SVEngine's accuracy by simulating genome-wide structural variants of NA12878 and a heterogeneous cancer genome. Our evaluation included checking various sequencing mapping features such as coverage change, read clipping, insert size shift, and neighboring hanging read pairs for representative variant types. Structural variant callers Lumpy and Manta and tumor heterogeneity estimator THetA2 were able to perform realistically on the simulated data. SVEngine is implemented as a standard Python package and is freely available for academic use .

## Background

Next-generation sequencing (NGS) has enabled researchers to detect and resolve complex genomic structural features at base-pair resolution. One can detect a variety of structural variations (SVs) including deletions, insertions, inversions, tandem duplications, and translocations based on NGS whole-genome sequence data [1]. A variety of algorithms have been developed for structural variant calling from NGS data. This includes programs such as Breakdancer, CNVnator, Delly, Haplotype Caller, Manta, Lumpy, SWAN, and Pindel, among others [2-10]. Even with these programs, accurate SV detection remains a significant challenge. For example, some SVs occur in lower allelic fractions as seen in tumors with intratumoral heterogeneity [11]. This is frequently the case as seen in genome sequencing of tumor samples, where cancer starts from a seeding clone and, through clonal evolution, successively acquires additional rearrangements at lower allelic fractions.

Benchmarking structural variant callers with available ground truth datasets is critical for software tool development, bioinformatics pipeline testing, and objective assessment of detection accuracy [12]. Whole-genome datasets are available from high sequencing coverage with Illumina or Pacific Bioscience systems [13]. However, for those users who wish to generate new sequencing datasets with specific features, identification and generation of ground truth datasets are laborious and cost prohibitive. Moreover, it is extremely difficult to empirically determine the analytical consequences of different sample processing methods, experimental variability in library preparation, and issues of sequencing bias in analysis [14].

Simulating NGS data provides an inexpensive alternative for assessing new algorithms in the context of sequencing data variation as noted in [15]. With simulated datasets, one can start refining analysis procedures *in silico*. Simulated NGS datasets can incorporate the variability associated with NGS sequence data, including sequencing coverage, number of libraries and insert size, base error rates, and tool parameters at the data analysis level. For *in silico* NGS data, a large number of SV characteristics can be readily designed, including the number, category, size, breakpoint sequence, variant fraction, and haplotype for any given locus. As a result, investigators can use this simulated data to assess the potential performance and make the trade-off between analysis cost and sensitivity before even carrying out the experiment.

Several programs generate NGS read sets to simulate metagenomics, or single nucleotide polymorphisms are available [16-22]. Only recently have we seen the development and release of structural variant simulators. An early example is RSVSim [23], an R package that amends template sequence files with structural variant changes. However, this program requires an interactive R session and, as a result, does not support batch processing. SCNVSim [24] improves on RSVSim by providing a command line interface. It simulates somatic copy number variants given a number of desired SV events and/or contigs. Nonetheless, both SCNVSim and RSVSim produce very limited variant-containing contig files (FASTA), which require external steps to simulate sequence reads (FASTQ) and output resulting alignments (BAM). VarSim [14] improves on RSVSim and SCNVSim with integrated read simulation using read simulators such as ART [25]. Instead of using a template sequence file, BAMSurgeon [26] patches an existing alignment file to embed structural variants. However, this application requires a high depth of coverage in the existing BAM file to successfully assemble a local contig for sequence patching. Moreover, the resulting structural variant may not have the exact breakpoints for the intended simulation. Overall, none of the listed tools provide a straightforward, joint control of an individual variant, including its exact breakpoints, ploidy, and locus-specific allelic fraction. These more complex features are particularly useful in simulating the clonal expansion of somatic structural variants, as seen in tumors.

As a solution to the limitations of current structural variant simulators, we designed and implemented SVEngine, a full-featured simulation program suite. SVEngine is capable of generating short sequence read sets, such as produced by an Illumina system, for thousands of spike-in variants that cover different types, sizes, haplotypes, and allelic fractions. Our application produces these simulated NGS datasets in a fraction of the time of other similar tools. SVEngine's flexibility for accepting different formats enables a user to generate whole-genome or targeted sequencing data that mimic germ-line, somatic, and complex clonal structured genomes with ease. It offers a high degree of allelic control through its parallelized divide-and-conquer planning scheme. In the simplest mode, users only need to provide the template (reference) sequences and a desired meta-distribution of type, size, and variant frequency in order to receive a full set of resulting FASTA, FASTQ, and BAM outputs along with the ground truth BED file.

### SVEngine features and simulation performance

We compare the available features of SVEngine with other simulators that include RSVsim [23], SCNVsim [24], VarSim [14], and BAMsurgeon [26], as shown in Table 1. SVEngine and the other tools can simulate common types of copy number events, e.g., deletions and tandem duplications. All simulators except SCNVsim simulate copy number neutral events, including insertions, inversions, and translocations. SVEngine improves the simulation of more complex SV events; it incorporates a variety of additional structural variant types originating from a combination of changes, such as inverted translocations, inverted duplications, duplicated translocation, and foreign sequence insertions. Users directly specify these events while preparing their input parameters; this process is more streamlined compared to other tools. For example, viral genome sequence insertion, which is a hallmark of the genomes of infected cells as seen in viral diseases and cancers [27], is easily achieved with SVEngine but not available with other simulation software except for BAMSurgeon.

**Table 1:** Available features of structural variant simulators

Use cases	SVEngine	RSVsim	SCNVsim	VarSim	BAMsurgeon
Copy number events: deletions, tandem duplications	✓	✓	✓	✓	✓
Copy number neutral events: inversions, insertions, translocations	✓	✓	✗	✓	✓
Phylogenetic clonal structure: cancer clonal evolution tree model	✓	✗	✓	✗	✗
Foreign sequence insertion: virus integration	✓	✗	✗	✗	✓
Non-human genome: variable haploid template and ploidy	✓	✓	✓	✗	✓
Not requiring pre-existing alignment: without BAM input	✓	✓	✓	✓	✗
Generate simulated contig: with FASTA output	✓	✓	✓	✓	✓
Generate simulated reads: with FASTQ output	✓	✗	✗	✓	✗
Generate simulated alignment: with BAM output	✓	✗	✗	✗	✓
Locus-specific variant ploidy: allelic imbalance	✓	✗	✗	✗	✗
Locus-specific variant frequency: variable somatic allele frequency	✓	✗	✗	✗	✓
Exact breakpoint: specifiable at base pair resolution	✓	✓	✗	✗	✓
Multiple sequencing libraries: e.g., multiple insert size, read length	✓	✗	✗	✗	✗

**Figure 1:**
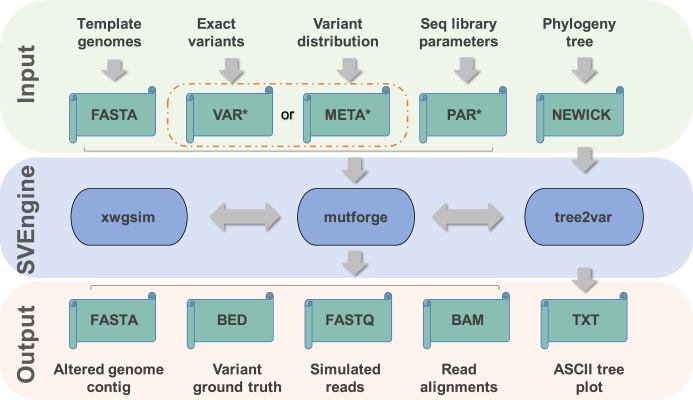
Inputs, outputs, and execution components of SVEngine. The flow of data is marked by gray arrows. The input, SVEngine functioning, and output data spaces are color shaded. *New file formats VAR, META, and PAR were introduced by SVEngine for specifying specific variants (VAR) or variants’ meta-distribution (META) to be simulated, or for specifying parameters for sequencing library and run (PAR). Please see the online manual for detailed explanations.

In terms of input/output flexibility and ease of use, SVEngine provides automation of template sequence modification, read simulation, and read mapping steps. These features are not found in other simulators of SV events. Also, SVEngine is the only tool that outputs a full set of simulation results in standard formats, including altered contig sequence (FASTA), simulated short reads (FASTQ), and alignment (BAM) files (Fig. 1). At the input step, all tools take in template sequences in FASTA format as the starting material, while BAMsurgeon additionally requires a pre-existing alignment file in BAM format as input. Overall, read coverage of this BAM file has to be large (typically >30x) in order to successfully assemble local contigs. Such requirements preclude the use of BAMSurgeon in applications generating low coverage and consequently limit its users to mimicking conditions based on available high-coverage BAMs. The VarSim tool needs structural variant prototypes from DGV [28], making it only applicable to the human genome. At the output step, RSVsim and SCNVSim provide modified sequence contigs in FASTA files. BAMsurgeon mainly outputs modified alignment in BAM files. The associated contigs need to be extracted from log files. VarSim provides both contigs containing a variant and simulated short reads, but it still requires additional user effort to generate alignment files.

With regard to precise and versatile control of individual variants, SVEngine enables one to easily specify variant type, size, exact breakpoint, ploidy, and allelic fraction for individual loci. Additionally, SVEngine simulates a full spectrum of germline, somatic, and clonal structural variations by the specified meta-distribution. In comparison, RSVSim does not support loci-level control, as it only patches template sequence on demand. With SCNVsim and VarSim, one only controls a meta-distribution of structural variants, such as the total number for each variant type and minimum and maximum variant size. SCNVsim allows the specification of ploidy, number, and type of clones but does not have the capability to specify exact breakpoints. VarSim randomly resamples breakpoint and other variant information from a DGV database dump. Only BAMsurgeon and SVEngine support locus-specific variant fractions, i.e., allowing different allele fractions for individual variants. Moreover, only SVEngine supports locus-specific ploidy, i.e., allowing a different ploidy state for individual variants. Both BAMsurgeon and SVEngine also support exact breakpoints for individual variants. However, in practice, the actual breakpoints generated by BAMsurgeon may differ from input as a result of improvised local contig assembly. Another unique feature of SVEngine is the ability to specify multiple sequencing libraries, which can each have different insert size mean and standard deviation, intended coverage depth, and read length.

In addition to the features listed in Table 1, SVEngine allows users to designate some regions while avoiding others. Examples of such applications include simulating exome or targeted sequencing datasets. This feature enables one to avoid complex regions such as telomeres and centromeres. SVEngine also features parallelized simulation by dividing a genome into pieces, embedding variants into each piece, and then stitching them together. Therefore, its performance can be boosted using the multicore processors.

Table 2 lists the runtime on a test set of  15,000 SV events into a 30x coverage whole-genome sequencing simulation, including 2,500 that consist of deletions, tandem duplications, inversions, translocations, and domestic or foreign sequence insertions. In multiprocessor mode, SVEngine has the shortest runtime in all three levels of simulation, i.e., obtaining altered contigs, simulated reads, and alignments in FASTA, FASTQ, and BAM formats in less than 10 minutes, 20 minutes, and 3 hours, respectively. Overall, SVEngine is 1x, 15x, and 48x times faster than the single-process SVEngine run. The performance scales almost linearly with the added central processing unit (CPU) power in generating the alignment output, because the read mapping time cost dominates other time costs, including data serializing time. Finally, even the single-process SVEngine (SVE-single) is more efficient than its other counterparts. For example, it took only 10 minutes for SVE-single to generate all altered contigs, while RSVSim and SCNVSim took several hours. SVE-single required half the time BAMSurgeon needs to generate all read alignments. All runtimes were measured on a computer server with four Intel Xeon E7-4850 CPUs (16 cores for each CPU) and with 256 GB shared random access memory (RAM).

**Table 2:** Runtime performance comparison

15,000 events at 30x coverage	SVEngine (64 cores)	SVEngine (1 core)	RSVSim	SCNVSim	VarSim	BAMSurgeon
FASTA output	<10 minutes	<10 minutes	10 hours	2 hours	Not available	Not available
FASTQ output	<20 minutes	5 hours	External	External	6 hours	Not available
BAM output	2 hours	5 days	External	External	External	>10 days

Not available: output format is not available.

External: output format is only available through additional external software, thus not tested.

### Simulating cancer genome evolution

**Figure 2:**
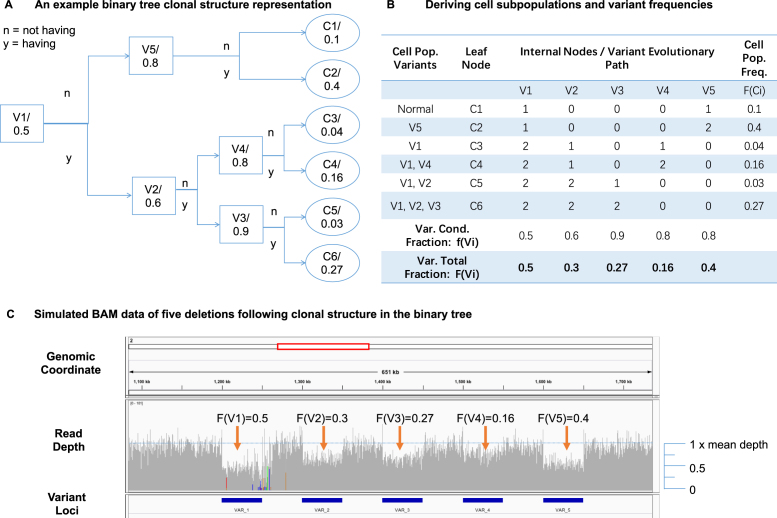
Simulating cancer evolution. **(A)** An example cancer evolution tree. The conditional fraction in each internal node represents the fraction of cell population gaining the next structural variation, which is represented by the label of the internal node. **(B)** An example computation table to determine final variant frequency of each variation and cell population frequency of each terminal genotypes. **(C)** Integrative Genomics Viewer view of SVEngine simulated BAM data of five deletions following clonal structure in the example binary tree.

SVEngine provides a high degree of control over SV events with variable allelic fractions. This feature enables one to simulate heterogeneous cancer genomes undergoing a phylogeny tree-structured clonal evolutionary process. As a demonstration, we present an example simulated with SVEngine (Fig. 2). To simplify the description of the phylogenetic process of cancer evolution, we use a binary tree representation of phylogeny. This binary tree is easily converted to a typical phylogeny tree by merging all nodes of identical cell subpopulations.

One example is a binary tree shown in Fig. 2A, where each of the five (internal tree nodes denotes a bifurcation event when part of the parental cell population is gaining an additional mutation (${V_j}, j = 1 \ldots m)$. The root node represents the lowest common ancestor cells of all subpopulations of cancer cells. These are typically normal cells that carry a genome that matches a germline genome, subsequently from which somatic genetic alterations accrue as part of cancer development. The root cell populations are split by the next immediate event, i.e., gaining the mutation ${V_1}$, resulting in two daughter cell populations depending on a cell's status of carrying ${V_1}$ or not, as represented by its two daughter cellular node. We denote the conditional cell fraction of gaining ${V_1}$ as $f( {{V_1}} )$, which is 50% or 0.5, in this case, and is denoted at the root. The mutational process goes on for subsequent internal nodes and until all variants (a total of five in this example) are represented by their bifurcation internal node. The resulting binary tree has six ($n = 6$) leaf nodes (${C_i}, i = 1 \ldots n)$, which represent all possible somatic genotypes of the terminal cell subpopulations.

As we can see, any terminal somatic genotype is completely determined by following the mutational path from the root down to a leaf node. We use a tertiary vector ${C_i} = ( {{c_{i,1}} \ldots {c_{i, m}}} )$ to indicate such a path, where

\begin{eqnarray*}
{c_{i,j}} = \left\{ {\begin{array}{lll}
{0, {\rm if}\ {V_j}\ \text{is not in the mutation path to}\ {C_i}} \\ 
{1, {\rm if}\ {V_j}\ \text{is in the mutation path to}\ {C_i}\ \text{but}\ {C_i}\ {\rm{doesn't}}\ {\rm carry}\ {V_j}} \\ 
{2,\text{if}\ {V_j}\ \text{is in the mutation path to}\ {C_i}\ \text{and}\,{C_i}\ \text{does carry } {V_j}}
\end{array}} \right.
\end{eqnarray*}

In addition, we define the conditional frequency $f( {{V_i}} )$, which is the fraction of cells derived from a parent population carrying event ${V_i}$ as: $f\ ( {{V_i}} ) = \frac{{\# \ {\rm{Child\ cells\ Gains}}\ {V_i}}}{{\# \ {\rm{Parent}}\,{\rm{cells\,at\,the\,verge\,of\,gaining\,}}{V_i}{\rm{\,}}}}$. Therefore, the final population frequency $F( {{C_i}} )$ of cell subpopulations ${C_i}$ is expressed as:

(1) \begin{eqnarray*}
F({C_i}) = {\rm{\,}}\mathop \prod \limits_{j:{c_{i,j}} >0} \left[ {\left( {{c_{i,j}} - 1} \right)*f\left( {{V_i}} \right) + \left( {2 - {c_{i,j}}} \right)\left( {1 - f\left( {{V_i}} \right)} \right)} \right]
\end{eqnarray*}

With $I( . )$ as the indicator function, the concurrent proportion $F( {{V_j}} )$ of all extant cells is simply the marginal sum of all cells carrying ${V_j}$:

(2) \begin{eqnarray*}
F({V_j}) = \mathop \sum \limits_{i = 1}^n I({c_{i,j}} = \,2)F({C_i})
\end{eqnarray*}

Fig. 2B shows the derivation of the above quantities for the example binary tree. The sequence of events ensures a partial order that the mutant allele frequency is always higher for events occurring upstream, as compared to events occurring downstream on the same lineage. It is possible that terminal genotypes may not all coexist in extant populations. The proposed binary tree representation accommodates a deceased population by having zero proportion for such a leaf node. SVEngine allows the user to input a binary tree with relevant bifurcation fractions to structure the variant fractions that fall along the line of the evolutionary tree. For designating this feature, the input to SVEngine is in standard NEWICK format, which is a widely accepted format that uses parentheses to encode nested tree structures [29]. Each internal node is labeled by the population splitting variant and weighted by the conditional splitting fraction. Each leaf node is labeled by associated terminal genotype and weighted by the subpopulation fraction as an optional feature. For instance, the NEWICK string for the example binary tree is: ((C1, C2) V5: 0.8, ((C3, C4) V4:0.8, (C5, C6) V3: 0.9) V2:0.6) V1:0.5.

Fig. 2C shows the Integrative Genomics Viewer view of SVEngine simulated BAM alignments of five equal-size deletions following the mutational process as represented by the example binary tree. The read depth shows the difference of allelic fractions corresponding to the computed final variant fractions based on the tree. We display an example of monoclonal cancer evolution, assuming that all cellular subpopulations start from a set of common ancestor cells (as denoted by the root node in the tree). In addition, simulations of multiclonal evolution are also possible with SVEngine. For example, one simply assigns an empty event to ${V_1}\,$ and then sets the conditional fractions of the two child events ${V_1}$ and ${V_5}$ to 100% to simulate a two-clonal origin evolution. With SVEngine's high efficiency, the simulation is easily scaled to tens of thousands of variants, with a tree having a more complex structure.

### Simulating the multitude of structural variations

**Figure 3:**
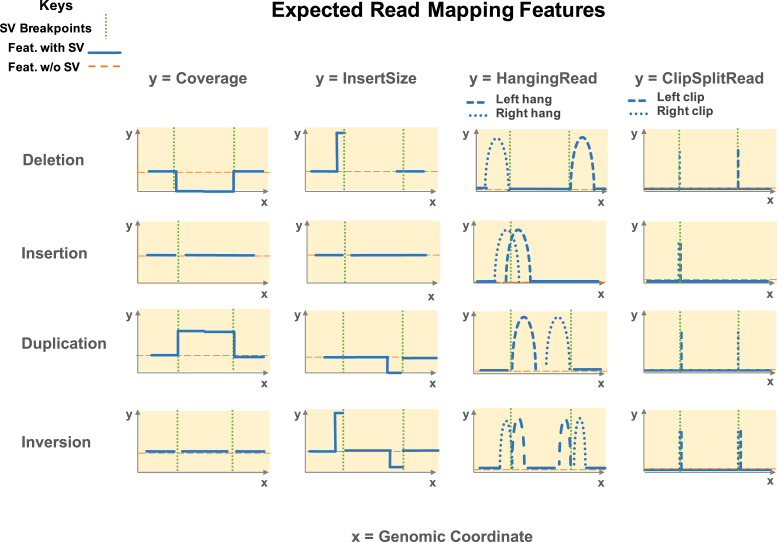
Expected read mapping features of structural variant prototypes. Rows—variant prototypes: 1) Deletion, 2) Insertion, 3) Duplication, 4) Inversion. Columns—mapping features: 1) Read coverage, 2) Read pair insert size, 3) Single end mapped read (HangingRead), 4) Soft clipped read or split mapped read (ClipSplitRead). The *x*-axis is genomic coordinates. The *y*-axis is feature value/counts. Dashed orange line stands for expected feature value without alteration. Solid blue line stands for expected feature value with alteration. Dotted green bar denotes the breakpoint(s).

Current structural variation detection methods mostly rely on detecting altered read mapping features to identify structure changes [30]. The most important features are read depth/coverage, read pair insert size, single-ended read pairs (hanging reads), soft-clipped reads, and split reads (clip/split reads). It is essential for structural variant simulators to correctly produce such feature changes corresponding to the causal event. In Fig. 3, we comprehensively illustrate the expected changes in mapping that result from different types of structural variants.

In the scenario of a deletion (Fig. 3, first row), all the mapping features such as coverage, insert size, hanging read and soft-clip/split read are expected to change, as illustrated in the *Coverage, InsertSize, HangingRead* and *ClipSplitRead* columns, respectively. First, there is a reduction of read coverage over the deleted region because no reads are present. Second, for those read pairs that are mapped straddling the breakpoints, the insert size is expected to increase as inferred by alignment to the reference. This extended insert size is possible because the deleted region is not present in the real DNA molecules where these read pairs originate from. Third, a fraction of read pairs aligning to the left of the left breakpoint lack a mapped mate read; this generates a right mate hanging read pair. This phenomenon is also called a right hang. This phenomenon occurs because the left breakpoint has interrupted the mate mapping by reducing similarity between the read and the reference. Due to symmetry, left mate hanging read pairs (left hang) form to the right of the deletion. Finally, when the breakpoint interruption in the mate is limited to the end of the read, it is possible that the mate read can still partially map. The noncontiguous part of the mate is either clipped or, if it is long enough, mapped to near the other end of the deletion. Such resulting read pairs are what we refer to as left or right soft-clipped (or split mapped) reads, depending on which side of the reads were split or clipped. These read pairs are expected to map right next to both breakpoints with the clipping (or splitting site) aligned to the exact breakpoint location, as shown.

For an insertion (Fig. 3, second row), the most noticeable change is the clustering of both the right- and left-hanging read pairs centering over the breakpoint. One observes a similar clustering for the left and right clip/split reads. As shown in Fig. 3, an insertion exhibits fewer changes than other types of structural variants, and so insertions are generally the most difficult to detect. In the scenario of a tandem duplication (Fig. 3, third row), the read coverage is expected to increase within the duplicated region. The insert size of reads mapping to the left of the right breakpoint is expected to decrease or even produce a negative value based on the duplication's position in the chromosome. This is the case because the mate is likely to have the same sequence as the segment preceding the read. Then, when the mate is mapped upstream of the current read, it causes a reversal of normal read strand order and introduces a negative insert size in the read mapping. By the same reasoning, the right-hanging, clipped, and split reads are clustered upstream next to the right breakpoint. Similarly, the left-hanging, clipped, and split reads are clustered downstream next to the left breakpoint, making the tandem duplication almost a mirror image of deletion.

In the scenario of an inversion (Fig. 3, fourth row), the coverage shows almost no change. The insert size near the left breakpoint is similar to the deletion scenario, which has an increase. This occurs as a result of the mate from the reverse complement of the other end of the inverted segment. This scenario creates an inflated insert estimate and an abnormal forward-forward strand read pair. Similarly, the insert size near the right breakpoint is decreased and forms an abnormal reverse-reverse strand read pair. When these abnormal pairs are interrupted by the breakpoints, it creates corresponding hanging read and clipped/split read clusters around both breakpoints. Citing another example, a chromosomal translocation is simply a combination of features at the region deleted by the translocation and insertion features at the region inserted.

### Simulation benchmark with NA12878

We additionally validated SVEngine's simulated data by applying popular structural variant callers and a tumor heterogeneity estimating tool to SVEngine-generated whole genome sequencing (WGS) data and benchmarked their performance with SVEngine's input ground truth. For our initial benchmark, we simulated WGS data for a well-studied individual NA12878 based on her known variants [31]. We applied the commonly used SV callers Lumpy [2] and Manta [10] and computed performance metrics such as the true-positive rate (TPR or sensitivity) and false discovery rate (FDR) for the callers at different simulated coverages. We simulated  20,759 structural variants for NA12878 with exact genotype and breakpoint information. The set included  19,034 deletions, 1,150 duplications, 328 inversions, and 247 insertions. These insertions included 91 LINE1, 58 ALU, and 9 SVA mobile element insertions, for which we determined the exact inserted sequence using RepBase [32].

**Figure 4:**
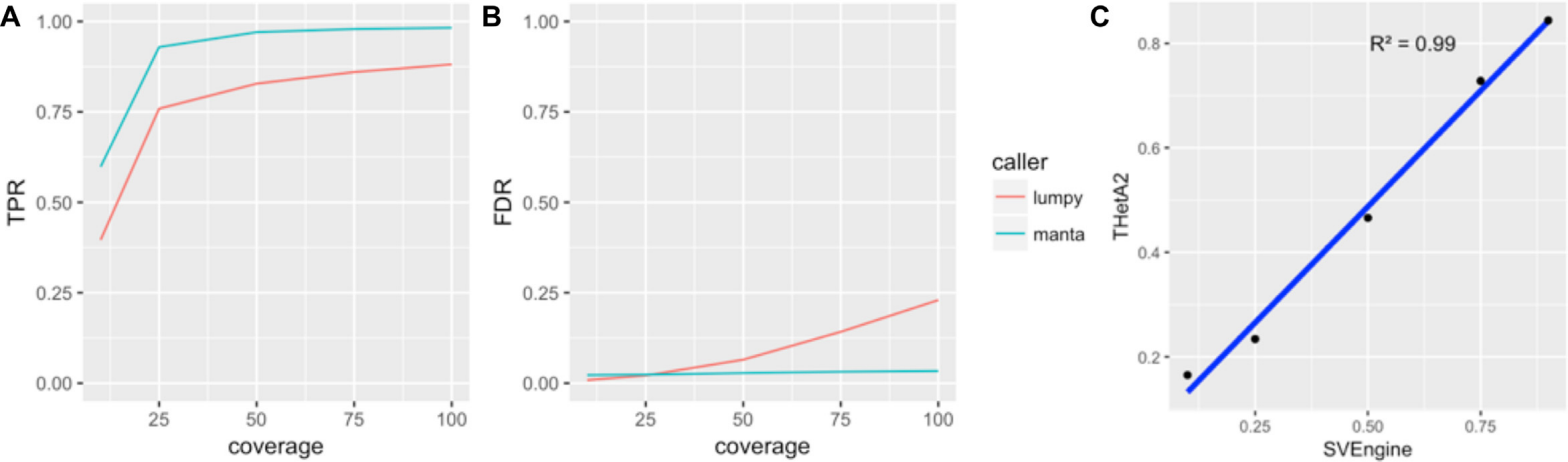
Simulation benchmark of SVEngine. We measured (**A**) TPR and (**B**) FDR for the two SV callers (Lumpy and Manta) using SVEngine simulated WGS data with 10x, 25x, 50x, 75x, and 100x coverage, respectively. (**C**) We measured the concordance in ${R^2}$ between THetA2 estimated purity and ground truth with 10%, 25%, 50%, 75%, and 90% tumor cell fractions based on SVEngine-simulated WGS data.

As shown in Fig. 4A and Fig. 4B, both SV callers performed well on the SVEngine simulated data. For Manta, the overall TPR ranges from 60% at 10x coverage to 98% at 100x. For Lumpy, the TPR is 40% at 10x coverage and rises to 88% at 100x. There seems to be a critical coverage value at around 25x, above which both callers can reach >80% sensitivity. This is in agreement with the widely accepted empirical coverage choice at 30x for WGS. The overall FDR for Manta was consistently within the 2%–3% range for average coverage, ranging from 10x to 100x. The FDR for Lumpy was considerably higher, from <1% at 10x to 23% at 100x. This is counterintuitive since we generally assume that higher coverage leads to improved performance and not the other way around. Why is this so for Lumpy? The deviation of the two tools in FDR may result from their different calling strategies. Manta likely has a dynamic coverage-based threshold. Lumpy's default parameter (as provided in its online manual) is likely tuned for <50x coverage for optimal performance. For higher coverage data, Lumpy may need to be calibrated, and this can be done using SVEngine's simulated data. As SV typing is still challenging for SV callers, to compute these overall TPR and FDR, we required only an adjacent match of predicted breakpoint and the ground truth.

If one considers the SV type (see Supplementary Table S1), deletions are the least challenging SV type for all callers. Manta TPR ranged from 60% to 99% as the coverage increased from 10x to 100x. Its FDR was consistently low at <1.2%. The same was true for Lumpy, which had a TPR increase from 42% to 88% with a consistently low FDR at <2%. Calling inversions were also well achieved by the callers. Manta and Lumpy TPR ranged from 83% to 100% and 66% to 97.3%, respectively, with increasing coverage. Both maintained an FDR that was approximately zero. Their performance diverged when it came to calling duplications and insertions. Manta maintained good performance at calling duplications, with TPR increasing from 47.5% to 90.6% with coverage and the FDR maintained close to zero. Its performance with insertion was less impressive, as the TPR increased from 0% to 41% with coverage with considerable FDR in the range of 30%–40%. On the other hand, Lumpy did not correctly type any duplications and insertions, and breakpoints of these SV types were more likely to be classified as unknown. Lumpy had a significantly increasing number of untyped calls with higher coverage. Between 30% and 50% of such calls were validated breakpoints of duplications and insertions. Generally, the SV callers performed well on calling and typing deletions, inversions, and duplications with SVEngine simulated data. Manta had the best overall performance. Calling and typing insertions were the most challenging task for SV analysis. SVEngine simulation of insertions will aid in further development of insertion callers.

### Simulation benchmark with tumor heterogeneity

To validate SVEngine's correctness in simulating various mutant allele frequencies, we simulated a series of WGS data representing two-population mixtures of tumor and normal genomes at a chosen level of tumor purity. We derived copy number segment files from the SVEngine simulated dataset and ran the tumor heterogeneity caller THetA2 [33] to infer the mixing subpopulation frequency.

We compare the THetA2 estimates to the input ground truth purity to SVEngine in Fig. 4C and Supplementary Table S2. The THetA2 estimated purities are (0.165, 0.234, 0.466, 0.728, and 0.844) for input ground truth purities at (10%, 25%, 50%, 75%, and 90%), respectively. The root mean square deviation is 0.043 and the Pearson correlation coefficient is 0.996 (i.e., ${R^2} >0.99)$). The high correlation and small error measure indicate SVEngine's correct simulation of tumor and normal mixture WGS data. The copy number segments derived from such data are suitable input to standard tumor heterogeneity tools such as THetA2 for reliably estimating tumor purities, even for simulating extreme cases where the tumor purity is as low as 10%.

## Conclusions

We have developed and released SVEngine, a structural variant simulator, available as an open-source program. It simulates next-generation sequencing data that has embedded structural variations as well as an assortment of complex sequence features. SVEngine simulates and outputs mutated sequence contigs (FASTA), sequence reads (FASTQ), and/or alignment (BAM) files with desired variants, along with BED files containing ground truth. SVEngine's flexible design enables one to specify size, position, and heterogeneity for deletion, insertion, duplication, inversion, and translocation variants. SVEngine's additional features include simulating sequencing libraries having multiple different molecular parameters and targeted sequencing datasets. SVEngine is highly parallelized for rapid and high-throughput execution.

We showed the versatility and efficiency of SVEngine by comparison of features and runtime vs. other available simulators. We demonstrated the utility of SVEngine in an example mimicking the phylogeny in cancer clonal evolution by simulating the associated variant allelic frequency. We validated the accuracy of SVEngine simulations by examining expected sequence mapping features such as coverage change, read clipping, insert size shift, and neighboring hanging read pairs for representative variant types. SVEngine is implemented as a standard Python package and is freely available for academic use [34].

The analysis of structural variants is an important part of genomics research. Improvements in the field also come from a growing set of available technologies, e.g., long read technologies such as the single-molecule, real-time sequencing by Pacific Biosciences [35]; nanopore sequencing by Oxford Nanopore Technologies [36]; and synthetic long read technologies such as the chromium droplet-based library preparations by 10X Genomics [37-39]. As the empirical data from these technologies accumulate, platform-specific read simulators such as PBSIM [40] and NanoSim [41] will become increasingly available. Although the implementation is nontrivial, the design of SVEngine is fully compatible with alternative read simulators. Going forward, we will work with the community to expand SVEngine with more powerful features, such as multiplatform simulation and cophased single-nucleotide polymorphism simulation.

## Methods

### Simulation software and pipeline

SVEngine was developed as a standard Python package with a C extension. SVEngine provides two Python executables and one C command line executable: *mutforge, tree2var* and *xwgsim*, respectively. The *mutforge* command implements a parallelized algorithm that divides the template genome into blocks of contigs, spikes structural variants into the contigs, samples short reads from the altered contigs, and finally merges the short-read sets back into one file and performs the alignment. The *tree2var* command implements a procedure that determines variant fractions from an input phylogeny tree based on Equations (1) and (2) and a depth first search graph algorithm and then substitutes these allele fractions in an input VAR file. The *xwgsim* command implements a modification to *wgsim*, which reduces the read sampling rate by 50% for the overlapping regions between contigs (i.e., ligation regions). The overlaps were designed so as to allow for the proper merging of contig-wise read sets. *xwgsim* only interacts with *mutforge* and thus is mostly transparent to a user.

As shown in Fig. 1, the required inputs to *mutforge* are three-fold: (1) a template haploid sequence file(s) in FASTA format. This can be a standard human genome reference or any other reference genome sequence. (2) A VAR file or a META file for specifying structural variants (distributions). These are tab delimited files with columns defined in SVEngine's manual. The VAR format is intended for specifying exact information for individual variants, which includes variant ID, parent ID (if part of a complex event such as a deletion occurring due to a translocation), fraction, ploidy, chromosome, starting position, and the sequence length to be deleted and/or the sequence content to be inserted. Alternatively, the META format is intended for higher-level control, allowing one to specify a desired meta distribution of variants, including variant type and total number of events, size, allele fraction, and ploidy distributions per type. One can specify where and how to insert the sample sequence in the case of foreign DNA insertion. For example, a user can readily design 100 deletions of size ranging from 100 bp to 10k bp of a uniform distribution of allelic fraction and a fair Bernoulli distribution of homo- and heterozygosity in one line of text in the META file. (3) The PAR file is used to model an experimental design, including insert size, read length, and coverage, as well as additional options for *xwgsim*. The file can be used to specify multiple libraries with different mean insert size and standard deviation. One can use such normal mixtures to approximate irregular libraries of multiple modes and asymmetric tails. The *xwgsim* command also provides random embedding of single nucleotide variations (SNV) and indels if desired. On the SVEngine's Wiki page, we supply example VAR, META, and PAR files with detailed annotation to facilitate their usage.

Once all inputs are provided, the SVEngine master process divides the template genome into blocks and serializes spike-in tasks to parallel worker processes. The worker process patches its assigned contig. If read pairs were required, it also calls *xwgsim* to simulate read pairs. The read pair subsets are then collected by the master process and merged. If alignments were required, it also calls *bwa-mem* and *samtools* to map the reads to the reference.

The output of SVEngine has three levels. At the first level (contig), only two files would be generated: one is a FASTA file containing all the altered contigs and the other is the ground truth of spiked-in variants in a BED3 format file with the additional columns following the VAR format as in the input. At the second level (read pair), SVEngine additionally outputs the read 1 and read 2 of the simulated read pairs in two FASTQ files. Finally, at the third level (alignment), SVEngine provides the read alignment output to the given reference in a BAM file format. The runtime of SVEngine increases with the specified output level, as additional processing time will be required. Table 2 can be used as a reference for runtime estimates for different output levels.

The *tree2var* command simulates a clonal evolution scenario, which requires an additional tree input file (NEWICK). *tree2var* also takes a VAR file, which can be generated from *mutforge* with a META file input and in the dry-run mode. The user must ensure that the identifier of the tree's internal nodes and the variant match each other, as this is used to identify and replace allele fraction with the value computed from tree phylogeny. The *tree2var* outputs a new VAR file that contains the rewritten allele fraction fields that reflect the clonal structure described by the user tree. For intuitive diagnostics, *tree2var* also outputs an ASCII text-based plot of the parsed input tree. SVEngine's tree parsing interacts with DendroPy [29], which allows further functionality such as random tree simulations and many tree statistics. The output VAR file from *tree2var* then becomes the input to *mutforge* for actual read simulation.

### A parallel simulation framework

**Figure 5:**
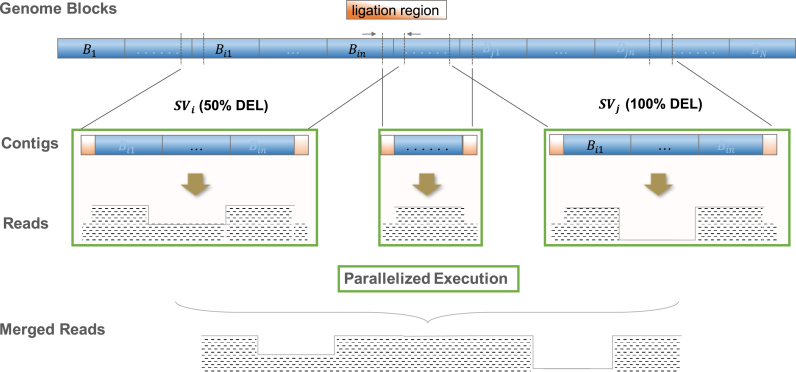
The core parallelized simulation algorithm of SVEngine. Here are two neighboring events $S{V_i}$ and $S{V_j}$: a 50% deletion and a 100% deletion to be spiked in. The first deletion event spans blocks ${B_{{i_1}}}$,${\rm{\,}}{B_{{i_1} + 1}}$, …,${\rm{\,}}{B_{{i_n}}}$, and the second deletion event spans genome blocks ${B_{{j_1}}}$,${\rm{\,}}{B_{{j_1} + 1}}$, …,${\rm{\,}}{B_{{j_n}}}$. The genome blocks are shaded in blue, and the ligation regions are shaded in orange. The resulting read pairs are represented by their coverage in black dash patterns. The parallel execution tasks are boxed in green.

SVEngine's major improvements to existing structural variant simulation tools involve one's ability to alter the allelic fraction, control of haplotypes, and highly efficient parallelized simulation. These improvements were achieved through the core algorithm as illustrated in Fig. 5. In general, we used a divide-and-conquer approach intertwined with multiprocess execution. First, the SVEngine master process lays out a genome grid for simulation. For any input haploid sequence, the entire genome is partitioned into $N$ equal size nonoverlapping blocks: ${B_1}$,${\rm{\,}}{B_2}$, …,${\rm{\,}}{B_N}$, where ${B_k} = \,[ {\frac{{G( {k - 1} )}}{N} + 1,\,\frac{{Gk}}{N}} ]$. The planned block size $\frac{G}{N}$ (i.e., plan size in the manual) can be chosen at the input, where $G$ is the entire genome length. Ligation regions of length $l$ (i.e., ligation size in the manual) are also defined, which consists of symmetric touching border regions of equal size adjacent blocks: ${L_1}$,${\rm{\,}}{L_2}$, …,${\rm{\,}}{L_{N - 1}}$, where ${L_k} = \,[ {\frac{{Gk}}{N} - \frac{l}{2} + 1,\,\frac{{Gk}}{N} + \frac{l}{2}} ]$. These serve as buffer regions that enable the SVEngine to ligate block sequence-based simulations back together. The block generating procedure is similar for multichromosome genomes, except blocks representing chromosome ends might be shorter than the standard block size.

Second, the SVEngine master process coordinates all of the tasks. In one task, a structural variant is embedded into the adjacent sequence. This is done by assigning a sequence of blocks that it impacts. All the variant's control information is attached to the task as well. In Fig. 5, the first variant, a 50% deletion $S{V_i}$, was assigned blocks ${B_{{i_1}}}$,${\rm{\,}}{B_{{i_1} + 1}}$, …,${\rm{\,}}{B_{{i_n}}}$, and the next variant, a 100% deletion $S{V_j}$, was assigned blocks ${B_{{j_1}}}$,${\rm{\,}}{B_{{j_1} + 1}}$, …,${\rm{\,}}{B_{{j_n}}}$. Depending on its size, a variant can take anywhere from one block to as many blocks as needed. The genomic region that is not altered between adjacent variants, e.g., $S{V_i}$ and $S{V_j}$, also becomes a task. This is assigned to the sequence blocks that are complementary to the blocks taken by $S{V_i}$ and $S{V_j}$ and with a no-op instruction attached. If necessary, no-op tasks with large block sequences are further broken down to no-op tasks with size-capped block sequences to improve efficiency of parallelization. The size cap is defined by the trunk size option as explained in the manual.

Third, the SVEngine master process dispatches all the tasks to an auto revolving worker process pool and then waits for all the workers to finish. Each worker process, when assigned a new task, loads the haploid sequence defined by the task's block sequence plus left and right ligation regions. For example, a worker would load sequence from $[ {\frac{{G{i_1}}}{N} - \frac{l}{2} + 1,\,\frac{{G{i_n}}}{N} + \frac{l}{2}} ]$ for $S{V_i}$ as the original contig, or $[ {\frac{{G{j_1}}}{N} - \frac{l}{2} + 1,\,\frac{{G{j_n}}}{N} + \frac{l}{2}} ]$ for $S{V_j}$, or $[ {\frac{{G({i_n} + 1)}}{N} - \frac{l}{2} + 1,\,\frac{{G( {{j_1} - 1} )}}{N} + \frac{l}{2}} ]$ for the no-op task in between them. The original contig is then operated on for deletion, insertion, or other alternations to form the altered contig. If no-op, the original contig is unaffected. The worker then calls *xwgsim* to simulate the proper numbers of read pairs from the original and altered contigs according to the specified frequency and resulting contig sizes. The *xwgsim* step also takes care of attenuated sampling (at half the normal rate) within the designated ligation regions as the worker provides the ligation size $l$ in its arguments. In addition, *xwgsim* adds a procedure to the popular NGS simulator *wgsim* [42] to correctly adjust coverage for a ligation region. Briefly, we define the ligation region as a segment of haploid sequence where two adjacent contigs to be simulated overlap. The ligation region is used to ensure proper and continuous transition from simulating reads from the first contig to the second. That also means a ligation region would be simulated twice—once along with the first contig and then along with the second contig. To compensate for potential double coverage in a ligation region, we implemented an adjusted read generation procedure in *xwgsim*, which only simulates 50% of the intended read coverage within the region for one contig. Such patterns of expected read pair coverage from the $S{V_i}$, $S{V_j}$, and no-op tasks are illustrated in Fig. 5.

Fourth, when the worker processes are completed, the master process collects all simulated read pairs from all tasks and concatenates them into two final files, one for read 1 and the other for read 2. Also, it collects all original and altered contigs and concatenates them into one final sequence file. Finally, it performs read pair alignment to the reference genome using *bwa-mem* and *samtools*. This last step, although sequential in SVEngine, is already thread parallelized by other required programs such as the *bwa* and *samtools* tools [22, 43]. Patterns of expected read pair coverages after merging the $S{V_i}$, $S{V_j}$, and no-op tasks are also illustrated in Fig. 5. The described algorithm assumes one haploid for simplicity. For multi-ploidy, each haploid is handled in a similar way by the worker process except that the variant's haplotype status is also taken into consideration. Overall, this SVEngine's core algorithm is very efficient, as demonstrated by the runtime comparison, and is very versatile and accurate, as demonstrated by multiple example applications described here.

### Notable simulator features

To comprehensively evaluate structural variant callers, one may need a wide spectrum and large number of SV events. This range is more easily specified by distributions of variants rather than individual variants. SVEngine supports variant distributions as specified in the META format. The expansion of distributions to actual variants takes place in the master process before any spike-in. The distributions are expanded on a target genome sequentially by randomly picking the next event's start position from regions that can accommodate it. Afterward, it removes the impact region from the remaining available regions, and so on. Once all distributions are expanded, the master process returns a list of variant fulfills user's specifications and outputs them into a VAR file. The user can choose to run SVEngine in dry-run mode to stop the execution at this point and inspect the resultant variants. The user also has the option to continue the simulation to the end, which is equivalent to inputting the output VAR file into SVEngine for simulation in the next step.

To increase the sensitivity of SV detection, researchers may prepare multiple sequencing libraries with different molecular parameters for analysis. For example, different insert sizes enable the detection of a wider spectrum of detection of SVs [44]. Longer sequence read length can boost the performance of some callers that use remapping strategies [45]. A unique feature of SVEngine is its ability to simulate NGS data modeling of multiple libraries with different mean insert size and standard deviation, coverage, and read lengths. The feature is implemented within the worker process. When using a multilibrary task, SVEngine will call *xwgsim* multiple times to generate read pairs in accordance with the library specification.

SVEngine provides simulation data that target or mask specific genomic regions. This feature emulates targeted sequencing applications, such as exome sequencing and gene panel sequence data. It can be used to exclude problematic regions such as gaps and telomere and centromere regions of the reference template. One only needs to provide standard BED format files to SVEngine listing the regions to be masked or targeted by the simulated sequencing.

Databases such as DGV and other literature provide a list of known population variants. Tools such as RepeatMasker [46] provide extensive lists of known regions of human repeats and/or homolog sequences, with enrichment of structural variant breakpoints. Although not provided in our examples due to their varied formats, in principle, these population and repeat-mediated variants can be downloaded in general tab delimited formats such as BED or VCF files. Subsequently, these annotation formats are easily converted into an SVEngine VAR format input file using text processing utilities such as *awk* and *sed*. Using a VAR file generated in this way, SVEngine can easily embed these variants into simulation data.

### Simulation benchmark with NA12878

To validate SVEngine's correctness in simulating various structural variants, we simulated WGS data for a well-studied individual NA12878 based on her known variants, as published by the 1000 Genomes Project (1KG) [31]. Then, we ran commonly used SV callers Lumpy [2] and Manta [10] and computed performance metrics such TPR and FDR for the callers as an indication of our simulation correctness.

In detail, we downloaded 1KG's NA12878 final call set from its ftp site [47]. We also downloaded human mobile element sequences from RepBase [32] (version 23.02). After cleaning up inconsistent variants registered by multiple callers and those that were mobile element insertion without available RepBase sequences, we arrived at  20,759 structural variants for NA12878 with exact genotype and breakpoint information. The set included  19,034 deletions, 1,150 duplications, 328 inversions, and 247 insertions. These insertions included 91 LINE1, 58 ALU, and 9 SVA mobile element insertions, the inserted sequence of which we were able to determine from the RepBase.

We encoded the information of these variants, such as SV type, genotype, breakpoint, and inserted sequences, into a VAR format suitable for input to SVEngine. We specified the sequencing library with a mean insert size of 300 and the sequencing run with 2 × 150 bp paired-end reads and ran SVEngine to generate a series of WGS data in BAM files with coverage of 10x, 25x, 50x, 75x, and 100x. The latest version of Lumpy and Manta were downloaded from their GitHub site and installed. We applied them on the SVEngine-generated WGS data series with their default parameters. The resulting SV calls were parsed into BED files and compared with the ground truth VAR file using BEDTOOLS. We consider a true positive hit if a call's breakpoint is within 20 base pairs of ground truth. We compared caller's performance both with and without enforcing correct SV typing. The performance metrics TPR and FDR were calculated as follows

\begin{eqnarray*}
True\,Positive\,Rate\,\,\left( {TPR} \right) = \frac{{\# \,of\,True\,Positives}}{{\# \,of\,Ground\,Truth}}\,\,\%
\end{eqnarray*}

and

\begin{eqnarray*}
False\,Discovery\,Rate\,\,\left( {FDR} \right) = \frac{{\# \,of\,Calls - \# \,of\,True\,Positives}}{{\# \,of\,Calls}}\,\,\% .
\end{eqnarray*}

### Simulation benchmark with tumor heterogeneity

To further validate SVEngine's correctness in simulating various mutant allele frequencies, we simulated a series of WGS data representing different mixtures of tumor and normal genomes, ran the tumor heterogeneity caller THetA2 [33] on the simulated data-derived segment file, and compared the THetA2 estimates to our ground truth purities.

In detail, we downloaded and installed the latest version of THetA2 from its GitHub site. We simulated a two-subpopulation scenario, where one tumor cell population and one normal cell population were mixed. We used the example segmental intervals provided by THetA2 as the ground truth copy number status of the tumor cell population and the human reference genome for the normal cell population. The copy number file has 84 segments, including 45 neutral, 26 losses, and 13 gains. We accordingly encoded these copy number variants into a series of VAR files with allelic frequencies: 10%, 25%, 50%, 75%, and 90%. We used SVEngine to generate WGS data BAM files based on the series of VAR files, with mean insert size 300 bp, 2 × 150 bp paired-end reads, and 100x coverage. We used *featureCounts* [48] to compute the probe segment file suitable for input to THetA2 and ran THetA2 with default parameters to estimate the tumor heterogeneity. We regressed the THetA2 estimated purity over SVEngine ground truth purity to find the ${R^2}$ statistic with the lm function in R.

## Availability of source code and requirements

Project name: SVEngine.

Project home page: https://bitbucket.org/charade/svengine.

Operating system: Linux/Unix

Programming language: standard Python package with a C extension

Other requirements: GNU C Compiler or similar

License: BSD3

Research Resource Identifier (RRID): SVengine, SCR_01 6235

## Availability of supporting data

In silico datasets are available via Bitbucket [34]. An archival copy of the Bitbucket repository is also available via the *GigaScience* database GigaDB [49].

## Additional files

**Table S1** Simulation benchmark on NA12878. The table includes full data of NA12878 simulation results, including computed true positive rate (TPR) and false discovery rate (FDR) for overall performance when SV typing correctness was not enforced (“all”) or for performance involving specific SV categories when SV typing correctness was enforced (“del”, “ins”, “inv”, “dup”).

**Table S2** Simulation benchmark on tumor heterogeneity. The table includes THetA2 estimated purity and input ground truth purity to SVEngine for the tumor heterogeneity simulation.

## Abbreviations

1KG: 1000 Genomes Project; CPU: central processing unit; FDR: false discovery rate; NGS: next-generation sequencing; RAM: random access memory; SV: structural variation/variant; TPR: true-positive rate; WGS: whole genome sequencing.

## Competing interests

The authors declare that they have no competing interests.

## Funding

(U01CA15192001) (R01HG006137) L.C.X., N.R.Z., and H.P.J. were supported by the National Institutes of Health (NIH) . N.A. was supported by the National Cancer Institute's (NCI) Cancer Target Discovery and Development Consortium (U01CA17629901), the NCI (K99 CA215256), and the Don and Ruth Seiler Fund. H.P.J. was also supported by the NIH (P01 CA91955). H.J.L. and H.P.J. were supported by the NIH . D.M.A. and C.L. were supported by the National Natural Science Foundation of China (61370131). Other support for H.P.J. came from the Gastric Cancer Foundation and the Research Scholar Grant (RSG-13-297-01-TBG) from the American Cancer Society.

## Author contributions

L.C.X., N.R.Z., and H.P.J. designed the study. L.C.X. developed the algorithm, wrote the program, and tested the software. H.J.L. and N.A. provided feedback on the software design and testing. L.C.X. and D.M.A. generated and analyzed the data with assistance from C.L. L.C.X. and H.P.J. wrote the manuscript. All authors approved the manuscript.

## Supplementary Material

GIGA-D-18-00023_Original_Submission.pdf

GIGA-D-18-00023_Revision_1.pdf

Response_to_Reviewer_Comments_Original_Submission.pdf

Reviewer_1_Report_(Original_Submission) -- Shengjie Gao, Ph.D 2/18/2018 Reviewed

Reviewer_1_Report_(Revision_1) -- Shengjie Gao, Ph.D 6/3/2018 Reviewed

Reviewer_2_Report_(Original_Submission) -- Adam Ewing 2/26/2018 Reviewed

Reviewer_2_Report_(Revision_1) -- Adam Ewing 6/4/2018 Reviewed

Reviewer_3_Report_(Original_Submission) -- Ryan Layer 2/27/2018 Reviewed

Reviewer_3_Report_(Revision_1) -- Ryan Layer 5/29/2018 Reviewed

Supplemental Tables
