# Supplementary material for: SVEngine: an efficient and versatile simulator of genome structural variations with features of cancer clonal evolution
Source: Gigascience. 2018 Jul 5;7(7):giy081. doi: 10.1093/gigascience/giy081 (PMC6057526; doi:10.1093/gigascience/giy081)
Supplement: GIGA-D-18-00023_Revision_1.pdf [file giy081_giga-d-18-00023_revision_1.pdf]

GigaScience

SVEngine: an efficient and versatile simulator of genome structural variations with features of cancer clonal evolution

--Manuscript Draft--

**Manuscript Number:**	GIGA-D-18-00023R1
**Full Title:**	SVEngine: an efficient and versatile simulator of genome structural variations with features of cancer clonal evolution
**Article Type:**	Technical Note
**Funding Information:**	National Human Genome Research Institute (P01 CA91955) — Dr. Hanlee P Ji<br>National Human Genome Research Institute (R01HG006137) — Dr. Hanlee P Ji<br>National Cancer Institute (U01CA15192001) — Dr. Hanlee P Ji<br>American Cancer Society (RSG-13-297-01-TBG) — Dr. Hanlee P Ji
**Abstract:**	Background: Simulating genome sequence data with variant features facilitates the development and benchmarking of structural variant analysis programs. However, there are only a few data simulators that provide structural variants in silico and even fewer that provide variants with different allelic fraction and haplotypes.<br>Findings: We developed SVEngine, an open source tool to address this need. SVEngine simulates next generation sequencing data with embedded structural variations. As input, SVEngine takes template haploid sequences (FASTA) and an external variant file, a variant distribution file and/or a clonal phylogeny tree file (NEWICK) as input. Subsequently, it simulates and outputs sequence contigs (FASTAs), sequence reads (FASTQs) and/or post-alignment files (BAMs). All of the files contain the desired variants, along with BED files containing the ground truth. SVEngine's flexible design process enables one to specify size, position, and allelic fraction for deletions, insertions, duplications, inversions and translocations. Finally, SVEngine simulates sequence data that replicates the characteristics of a sequencing library with mixed sizes of DNA insert molecules. To improve the compute speed, SVEngine is highly parallelized to reduce the simulation time.<br>Conclusions: We demonstrated the versatile features of SVEngine and its improved runtime comparisons with other available simulators. SVEngine's features include the simulation of locus-specific variant frequency designed to mimic the phylogeny of cancer clonal evolution. We validated SVEngine's accuracy by simulating genome-wide structural variants of NA12878 and a heterogenous cancer genome. Our evaluation included checking various sequencing mapping features such as coverage change, read clipping, insert size shift and neighbouring hanging read pairs for representative variant types. Structural variant callers Lumpy and Manta and tumor heterogeneity estimator THetA2 were able to perform realistically on the simulated data. SVEngine is implemented as a standard Python package and is freely available for academic use at: https://bitbucket.org/charade/svengine.
**Corresponding Author:**	Hanlee P Ji, M.D.<br><br>UNITED STATES
**Corresponding Author Secondary Information:**	
**Corresponding Author's Institution:**	
**Corresponding Author's Secondary Institution:**	
**First Author:**	Li C Xia, PhD
**First Author Secondary Information:**	

Powered by Editorial Manager® and ProduXion Manager® from Aries Systems Corporation

**Order of Authors:**	Li C Xia, PhD
	Dongmei Ai, PhD
	Hojoon Lee, PhD
	Noemi Andor, PhD
	Chao Li
	Nancy R Zhang, PhD
	Hanlee P Ji, M.D.
**Order of Authors Secondary Information:**	
**Response to Reviewers:**	We thank the editors and reviewers offered their great suggestions and comments based on the previous version of SVEngine manuscript. With this revision, we addressed all the issues raised by the reviewers and editors. And through the process, we had improved the overall quality of this manuscript. We hope the revised manuscript achieves the high standard of scientific publication of GigaScience. Thank you for your consideration.<br><br>Sincerely,<br><br>Hanlee Ji, MD<br><br>Associate Professor of Medicine<br>Stanford University School of Medicine<br><br>[Point-to-Point Response]<br><br>[Response to editors' comments]<br>Among the major points of the reviewers I'd like to highlight the need for benchmarking and evaluation of the simulations, to show that the result resembles real data (See the comments of reviewers 2 and 3).<br><br>Response:<br>We have performed additional benchmark simulation as suggested by the two reviewers. In the revision, we reported additional simulation findings and released the data to reproduce our results in the public domain (https://bitbucket.org/charade/svengine/wiki/Example). By applying two popular SV callers and one tumor heterogeneity estimator to these data and studying their performance, we further confirmed the validity of our simulations. For details, please see our point-to-point<br>responses to reviewer #3' specific comments.<br><br>A minor comment that came up during editorial discussion of the paper was that ample development is currently going into SV discovery / assembly software based on long read technology (PacBio / ONT), and we wondered whether it would be possible to amend the software in the future for this purpose? (this is a discretionary comment that you can decide to discuss in the paper, or not).<br>Response:<br>We thank the editors for raising this point to us and fully understand its importance. We added following discussion to the end of this manuscript as copied below:<br>"Structural variant analysis is a significant component of genomics research, which is continuously being improved by a growing set of available technologies, e.g. long read technologies such as the single-molecule, real-time (SMRT) sequencing by Pacific Biosciences [35] and the nanopore sequencing by Oxford Nanopore Technologies [36], or synthetic long read technologies (SLR) such as the Chromium droplet-based library preparations by 10X Genomics [37, 38]. As the empirical data from these technologies accumulate, platform specific read simulators like PBSIM [39] and NanoSim [40] will become increasingly available. Although the implementation will be non-trivial, the design of SVEngine is fully compatible with alternative read simulators. Going forward, we will work with the community to expand SVEngine with more powerful features, such as multi-platform simulation and co-phased SNP simulation."<br>We noticed there are only a few currently published read simulators for Pacbio and

Powered by Editorial Manager® and ProduXion Manager® from Aries Systems Corporation

ONT technologies such as PBSIM and NanoSim. The design of SVEngine would allow them to be integrated. It does require some familiarity of each read simulator's source code to do the proper source-code level modification. Since SVEngine is being publicized open source, we expect us, with the help from read simulator developers, will be able to implement these requested features in the near future.

- I believe you had discussed software licensing with one of my colleagues at submission if I read the notes on the file correctly. As discussed, please change the (non-OSI) Stanford license to an OSI-license such as, for example, BSD3 (https://opensource.org/licenses/BSD-3-Clause).
Response:
Yes. We had discussed the licensing options with the GigaScience editor's. The SVEngine software is now released as OSI compatible BSD3.

- Please register your new software application in the SciCrunch.org database to receive a RRID (Research Resource Identification Initiative ID) number and include this in your manuscript. This will facilitate tracking, reproducibility and re-use of your tool.
Response:
Yes. We have registered SVEngine with SciCrunch and the status is curated. The associated RRID is SCR_016235.

If you are able to fully address these points, we would encourage you to submit a revised manuscript to GigaScience.
Minor comment from editorial discussion:- The authors write: It is typically the normal germline cell as depicted here, or the first generation of cells bearing common somatic mutations that start becomingcancerous. In fact it is not typically the germline cell itself that becomescancerous, but cells that carry a genome that (presumably) matches the genome of the germline.

Response:
We thank the editors for finding out this inaccuracy in our language. We have updated the manuscript to correctly convey the idea. The corrected sentence is:
"The root node represents the lowest common ancestor cells of all subpopulations of cancer cells. These are typically normal cells that carry a genome that matches a germline genome, subsequently from which somatic genetic alterations accrue as part of cancer development."

[Response to reviewer #1's comments]
Reviewer #1: This manuscript presents the design and implementation of a structural variant simulation software called SVEngine. Compared with related software, SVEngine provides new features, such as the simulation of locus-specific variant frequency. Parallelization optimization is also adopted to improve the computing speed of SVEngine. It is a topic of interest to the researchers in the related areas, but the paper needs minor reversion before acceptance for publication.
My comments are as follows:The author claimed the performance of SVEngine scales almost linearly with the added CPU power (1x, 15x and 48x times faster than the single-process run) in the last paragraph of page five. The configuration of the computing server mentioned in the article (first paragraph of page six) is a computer equipped with one Intel i7-4790 CPU, which has four physical cores and supports eight threads by hyper-threading. If the threads don't do I/O and there's nothing else running, one thread per core will get you the best performance. However, that very likely not the case in the experiment (48x speedup on 4 physical core). As the computation performance is one of the highlights of SVEngine, the explanation of the acceleration should be provided in detail. Evaluation and analysis under different parallel scale are also needed, so as to locate the performance bottleneck and support the parallel speedup of the pipeline.

Response:
We thank the reviewer for the positive and constructive comments. To the reviewer's question, we apologize the CPU and computer server information was incorrect in the previous version, which caused the confusion. We can confirm that the performance was measured on a 4-CPU computer cluster, where each CPU has 16 physical cores. In total there is 64 cores that together give us a 48x speedup. The acceleration is

almost linear and within our expectation. The corrected text is below:

"All run time were measured on a computer server with four Intel® Xeon E7-4850v4 CPUs (16 cores each CPU) and with 256 GB shared RAM."

To perform analysis under different parallel scales, we have provided additional benchmark data that supports SVEngine's efficient computational performance (see our newly added data for NA12878 and a cancer genome in the revised paper). In these experiments, we have successfully simulated ~ 20,000 in silico events in the entire human genome at 100X coverage without issues. To generate the final BAM file took about 1 day on a 32 physical cores (4 units of AMD Opteron 6386 SE, 8 physical cores each unit) and 256GB RAM server. We found the overall runtime was linearly bounded by the number of events being modeled and the total coverage and scaled nearly as a fraction of the available cores. We can identify the real step has the potential to be a bottleneck is to map the short reads toward human genome, which was actually performed with bwa. It is out the scope of SVEngine's design and is optionally substituted if necessary. We hope we can convince the reviewer, SVEngine is an efficient simulator, given that we have simulated dozens of human genomes with tens of thousands of all types of events and varied coverages without issues (these numbers are in realistic range for total SVs based on literature).

[Response to reviewer #2's comments]
Reviewer #2: SVEngine is a welcome addition to a niche of variant simulation tools that can produce structural rearrangements for the purpose of benchmarking SV detection tools. SVEngine provides a few features not found elsewhere, the most notable perhaps is the ability to simulate subclonality with a bifurcating tree model.
Given that this is a tool intended to be used for benchmarking, it would be helpful to see some benchmarking data in the manuscript to reassure the reader that SVEngine does indeed create SVs of each type supported that are detectable with standard SV calling tools.
Response:
We thank the reviewer for the suggestion. In the revision we have added substantial new benchmark data to further validate the performance of SVEngine using existing SV calling tools. Since the newly added benchmark was structured based on reviewer #3's first comment, we refer reviewer #2 to our response there. The first part of new benchmark data we described there addressed this comment.

What is the utility of supporting multiple insert sizes within the same simulation? How often, in recent practice, does one encounter a sequencing run that was constructed with multiple insert sizes?
Response:
Regarding the reviewer's question on the utility of multiple insert size library, we agree that such use is not common in our experience. However, there are circumstances where this occurs. For example, we know of experiments where it was intentionally designed three libraries of different insert sizes in the hope to perform sensitive and comprehensive detection for deletions. Their rationale was, for each insert size, the effective detection range is at least two standardard deviations away from the mean. So, the use of multiple insert size would allow the combined effective detection range to cover the full size spectrum of deletions. However, since that design was not prospectively validated with bioinformatics tools using simulation data, it had caused significant challenge in downstream analysis. Now with SVEngine, the pro and cons of multi-insert design, if desired, can be studied before sequencing.
Another example involves mate-pair sequencing where larger inserts, 2 kb or more are used to improve the detection of structural variants. To have benchmark in silico data sets to reflect the properties of these libraries would require varying the insert size.
The multiple insert size simulation is also useful retrospectively, in particular when multi-modal or asymmetric insert-size distributions arise from real data. In principle, such multi-modal or asymmetric distributions can be approximated by a mixture of normal densities. Using SVEngine to generate similarly distributed data with known ground truth will enable one to benchmark the performance and robustness of intended bioinformatics analysis. Note, this task would not be possible with the real data only because the ground truth would not be available. We thank the reviewer for asking this great question and we have added our answer to our online FAQ.

I think the items in Fig 1 could use a bit more explanation. For example, from the figure, 'sequencing library' might be interpreted to mean an actual fastq file, but it actually means a file with information on paired end read distributions as per the example on bitbucket, and "PAR" is just an arbitrary extension.
Response:
We thank the reviewer for the valuable suggestions. We have updated Figure 1 to accommodate them accordingly. The newly introduced file formats like VAR, META and PAR are starred. We briefly explained them in the figure caption and pointed readers to the online manual for additional information. The added caption text is copied here:
"(*) Note: new file formats VAR, META and PAR were introduced by SVEngine for specifying specific variants (VAR) or variants' meta-distribution (META) to be simulated, or for specifying parameters sequencing library and run (PAR). Please see the online manual for detailed explanations."

Page 15, lines 51-53 "In addition, xwgsim adds a procedure to the popular NGS simulator wgsim [31], which rejects a new read pair at 50% chance if any of its two ends originates in a ligation region." -- I wasn't able to work out why this is necessary, could you clarify?
Response:
We apologize for the insufficient description. We defined the ligation region as a segment of haploid sequence where two adjacent contigs to be simulated overlaps (see Figure 4). The ligation region is employed to ensure proper and continuous transition from simulating reads from the first contig to the second. That also means a ligation region would be simulated twice – once along with the first contig, and then along with the second contig. To compensate for potential double coverage in a ligation region, we implemented an adjusted read generation procedure in xwgsim, which only simulates 50% of the intended read coverage within the region for one contig. The modified text is copied here:
"Briefly, we define the ligation region as a segment of haploid sequence where two adjacent contigs being simulated overlaps. The ligation region is employed to ensure proper and continuous transition from simulating reads from the first contig to the second. That also means a ligation region would be simulated twice – once along with the first contig, and along with the second contig. To compensate for potential double coverage in a ligation region, we implemented an adjusted read generation procedure in xwgsim, which only simulates 50% of the intended read coverage within the region for one contig."

Is xwgsim integral to running SVEngine or could another read simulator be swapped in e.g. ART? I ask because wgsim is mainly aimed at Illumina data but simulators may exist for other data types and the ability to use them would extend the usefulness of SVEngine.
Response:
The short answer is yes. To make possible parallelized processing by SVEngine, in xwgsim, we modified the source code of wgsim to properly carry out contig-wise simulation employing ligation regions so as to precisely achieve desired simulation. xwgsim is thus integral to SVEngine.
Nonetheless, the source-code level modification as we did to wgsim is not complicated, which is likely also doable with other open source reader simulators and for other types of sequencing platforms, e.g., ART as mentioned here. It, however, does require some familiarity of the read simulator's source code. Since SVEngine is to be published open source, we expect, with help from read simulator developers, could implement such features, as the community getting actively engaged and coming up with these requests.
Similarly, it wasn't clear how configurable BAM generation was: suppose I want to use bowtie and not bwa or whatever aligner is the default - is this possible?
Response:
The short answer is yes. The direct BAM generation of SVEngine is currently tided to BWA MEM with default parameters. However, if that is not desired behavior, one can always take over after the FASTQ files have been generated and apply any available

Powered by Editorial Manager® and ProduXion Manager® from Aries Systems Corporation

aligner as wishes. So, what has been asked is not only possible but easily doable by setting the option '-x fastq' to SVEngine. It is actually our current default to generate FASTQ files as end points of simulation (please refer to our online manual). The simulated read pairs will be saved into two FASTQ files 'xxx.fq1' and 'xxx.fq2', which are suitable input to bowtie or other aligners of choice. We have included this Q&A information in our online FAQ.

This is referring to the program itself and not the paper: Is there an intuitive explanation for what 'trunksize' and 'plansize' mean?
Response:
Yes. We intuitively explained these terms below and the explanations were added to the online FAQ and the online manual.
Trunksize is a term we used to define the size of parallelly processed haploid contigs when no structural variant is present (non-variant contigs). The divide of non-variant contigs into trunks enables concurrent simulation of reads and their efficient merge with reads from variant contigs. The current default trunksize is 10 Megabase, which, if considering no variants to be simulated at all, SVEngine will distribute the overall simulation to around 300 trunks for the human genome. In practice, the parameter should be considered in conjunction with the number of processors available. Intuitively, if the number of processors is 30, and by the default trunksize 1MB, each processor will process around 10 trunks before finishing. In this case, the jobs are well distributed. But if the trunksize was chosen as 1 GB, then only around 3 trunks will be distributed parallelly and the rest 27 processors will be idling, which reduces SVEngine's efficiency and increases its running time, because it has to wait for the bigger sized (now 1GB) trunk simulation to finish. The rule of thumb is to choose trunksize as a fraction of what the genome size divided by the number of processors. The trunksize, however, does not affect the task distribution of variant contigs as these contigs are always created and distributed per variant basis.
Plansize is a term we used to describe the coarse grain unit size when we try to randomly distribute a desired number of structural variants genome-wide. It is needed when the input is a META file, which specifies a distribution of structural variants to be simulated. The current default is 100 Kilobase. Intuitively, for the human genome, the default is to divide it into around 300,000 units, all of which are marked available at the beginning. SVEngine then randomly samples from the available units to accommodate the next variant and mark the relevant units unavailable. The process continues until the desired distribution is achieved. For each variant, depending on its size and type, it could take a contiguous segment of one or more units. If the specified distribution turns out not accommodatable with current plansize, SVEngine will report an informative error and stop before any actual read simulation. One can then retry the simulation with reduced plansize or opt for a less ambitious simulation.

A few notes on the comparison with BAMSurgeon, the various points made are largely fair, but there are a few features the authors have missed. BAMSurgeon does support insertions including insertions of arbitrary sequences (e.g. viral sequences) through the INS type (see manual, pg 9-10). BAMSurgeon also does output the contigs generated before and after SV spike-in - they're in the addsv_logs_* directory after the run is complete. This isn't well-documented however. Finally, the user is able to specify per-variant allele fraction through the -c/--cnvfile option, although it is admittedly a bit arcane (page 4 of the manual has an explanation). These omissions are perhaps understandable to an extent but it raises the question of whether features have similarly been missed for the other tools compared to SVengine in this paper.
Response:
We thank the reviewers for pointing out the details regarding BAMSurgeon which we have overlooked. We acknowledge those valid points and have updated our comparison table to tick on the 'locus-specific variant frequency', 'foreign sequence insertion' and 'generate simulated contig' boxes for BAMSurgeon. We have rechecked publications and manuals of other tools and reaffirmed other comparisons made in the table.

[Response to reviewer #3's comments]
Reviewer #3: The authors present an SV simulation framework that is fast, easy to use, and the bitbucket documentation is good. Making heterogeneous SV datasets is very

Powered by Editorial Manager® and ProduXion Manager® from Aries Systems Corporation

	important to test new SV detection methods, and SVEngine makes this much easier.<br>I do have one major issue:<br>There needs to be an evaluation of the simulations to show that the result resembles real data. For a "normal" case, this could be as easy as simulating NA12878's SVs, then running a handful of SV callers on both the real and simulated data and show that the results are similar. Given the title of the paper, the authors also need to show that the clonal populations that were specified are observed in the data. Something like THetA (L. Oesper Bioinformatics, 2014) or one of its successors could be used here.<br>Response:<br>We first thank the reviewer for the positive and constructive feedbacks. As suggested by the reviewer, we included two additional set of benchmark data with this revision.<br>In the first benchmark:<br>“We additionally validated SVEngine’s correctness by applying popular structural variant callers and a tumor heterogeneity estimating tool to SVEngine generated WGS data and benchmarked their performance with SVEngine’s input ground truth. In the first benchmark, we simulated WGS data for a well-studied individual NA12878 based on her known variants [31]. We applied commonly used SV callers: Lumpy [2] and Manta [10] and computed performance metrics such true positive rate (TPR or sensitivity) and false discovery rate (FDR) for the callers at different simulated coverage. In total, we simulated 20759 structural variants for NA12878 with exact genotype and breakpoint information. The set included 19034 deletions, 1150 duplications, 328 inversions and 247 insertions. These insertions included 91 LINE1, 58 ALU and 9 SVA mobile element insertions, for which we determined the exact inserted sequence using RepBase [32].”<br>In the second benchmark:<br>“To further validate SVEngine’s correctness in simulating various mutant allele frequency, we simulated a series of WGS data representing two-population mixtures of tumor and normal genomes at a chosen grade of tumor purities. We derived copy number segment files from the SVEngine simulated data set and ran the tumor heterogeneity caller THetA2 [33] to infer the mixing subpopulation frequency.”<br><br>The methods and results details of these benchmarks were added and were highlighted in the revision. These include a newly added Figure 5 and two newly added Supplementary tables S1 and S2. In summary, commonly used SV callers, Manta and Lumpy, generally performed well on calling and typing deletions, inversions and duplications with SVEngine simulated data. Calling and typing insertions is so far the most challenging task for SV analysis and the capability of SVEngine to simulate various insertions can aid the further development of insertion callers. Also, the copy number profiles derived from SVEngine simulated tumor and normal mixture WGS data are suitable input to standard tumor heterogeneity tools such as THetA2 for reliably estimating tumor purities, as demonstrated by good correlation and small residual errors between THetA2 estimates and input ground truth purities.<br>The results from both benchmarks have further validated the correctness and efficiency of SVEngine and the inclusion of which has strengthened the manuscript greatly.<br><br><br>I also have a suggestion:<br>This method would be much more powerful if it could simulate a diploid (or more) genome with accompanying SNPs properly phased. I would expect clonal evolution methods to use both SNPs and SVs, and that phasing these events would be quite powerful. If SVEngine supported phased simulations, then it would have a wider audience.<br>Response:<br>We thank the reviewer with this great suggestion and agreed such simulation would be more powerful. Simulating SNPs and small indels require additionally domain knowledge and a tool encompassing both requires extensive benchmark work that together would surpass the scope of this manuscript. But we will definitely consider this suggestion in our future development of SVEngine.
**Additional Information:**	
**Question**	**Response**
Are you submitting this manuscript to a	No

Powered by Editorial Manager® and ProduXion Manager® from Aries Systems Corporation

special series or article collection?	
**Experimental design and statistics**<br><br>Full details of the experimental design and statistical methods used should be given in the Methods section, as detailed in our Minimum Standards Reporting Checklist. Information essential to interpreting the data presented should be made available in the figure legends.<br><br>Have you included all the information requested in your manuscript?	Yes
**Resources**<br><br>A description of all resources used, including antibodies, cell lines, animals and software tools, with enough information to allow them to be uniquely identified, should be included in the Methods section. Authors are strongly encouraged to cite Research Resource Identifiers (RRIDs) for antibodies, model organisms and tools, where possible.<br><br>Have you included the information requested as detailed in our Minimum Standards Reporting Checklist?	Yes
**Availability of data and materials**<br><br>All datasets and code on which the conclusions of the paper rely must be either included in your submission or deposited in publicly available repositories (where available and ethically appropriate), referencing such data using a unique identifier in the references and in the "Availability of Data and Materials" section of your manuscript.<br><br>Have you have met the above requirement as detailed in our Minimum Standards Reporting Checklist?	Yes

Manuscript
Click here to view linked References
Click here to access/download;Manuscript;svengine_manuscript_26_June1

SVEngine: an efficient and versatile simulator of genome structural variations with features of cancer clonal evolution

Li Charlie Xia[1,2], Dongmei Ai[3], Hojoon Lee[1], Noemi Andor[1], Chao Li[3], Nancy R. Zhang[2], Hanlee P. Ji[1,4,*]

[1]Division of Oncology, Department of Medicine, Stanford University School of Medicine, Stanford, CA 94305

[2]Department of Statistics, the Wharton School, University of Pennsylvania, Philadelphia, PA 18014

[3]School of Mathematics and Physics, University of Science and Technology Beijing, 30 Xueyuan Road, Haidian District, Beijing 100083 P. R. China

[4]Stanford Genome Technology Center, Stanford University, Palo Alto, CA 94304

* To whom correspondence should be addressed

genomics_ji@stanford.edu

Running Title:

SVEngine: simulation of structural variations for cancer clonal evolution

ABSTRACT

Background: Simulating genome sequence data with variant features facilitates the development and benchmarking of structural variant analysis programs. However, there are only a few data simulators that provide structural variants in silico and even fewer that provide variants with different allelic fraction and haplotypes.

Findings: We developed SVEngine, an open source tool to address this need. SVEngine simulates next generation sequencing data with embedded structural variations. As input, SVEngine takes template haploid sequences (FASTA) and an external variant file, a variant distribution file and/or a clonal phylogeny tree file (NEWICK) as input. Subsequently, it simulates and outputs sequence contigs (FASTAs), sequence reads (FASTQs) and/or post-alignment files (BAMs). All of the files contain the desired variants, along with BED files containing the ground truth. SVEngine's flexible design process enables one to specify size, position, and allelic fraction for deletions, insertions, duplications, inversions and translocations. Finally, SVEngine simulates sequence data that replicates the characteristics of a sequencing library with mixed sizes of DNA insert molecules. To improve the compute speed, SVEngine is highly parallelized to reduce the simulation time.

Conclusions: We demonstrated the versatile features of SVEngine and its improved runtime comparisons with other available simulators. SVEngine's features include the simulation of locus-specific variant frequency designed to mimic the phylogeny of cancer clonal evolution. We validated SVEngine's accuracy by simulating genome-wide structural variants of NA12878 and a heterogenous cancer genome. Our evaluation included checking various sequencing mapping features such as coverage change, read clipping, insert size shift and neighbouring hanging read pairs for representative variant types. Structural variant callers Lumpy and Manta and tumor heterogeneity estimator THetA2 were able to perform realistically on the simulated data. SVEngine is implemented as a standard Python package and is freely available for academic use at: https://bitbucket.org/charade/svengine.

Keywords: Structural variation, next generation sequencing, sequence analysis, locus-specific allele frequency, somatic haplotypes, cancer clonal evolution.

FINDINGS

Background

Next generation sequencing **(NGS)** has enabled researchers to detect and resolve complex genomic structural features at base-pair resolution. One can detect a variety of structural variations **(SVs)** including deletions, insertions, inversions, tandem duplications and translocations based on NGS whole genome sequence data [1]. A variety of algorithms have been developed for structural variant calling from NGS data. This includes programs such as Breakdancer, CNVnator, Delly, Haplotype Caller, Manta, Lumpy, SWAN, Pindel among others [2-10]. Even with these programs, accurate SV detection remains a significant challenge. For example, some SVs occur in lower allelic fractions as seen in tumors with intratumoral heterogeneity [11]. This is frequently the case as seen in genome sequencing of tumor samples, where cancer starts from a seeding clone and through clonal evolution, successively acquires additional rearrangements at lower allelic fractions.

Benchmarking structural variant callers with available ground truth data sets is critical for software tool development, bioinformatics pipeline testing and objective assessment of detection accuracy [12]. Whole genome data sets are available from high sequencing coverage with Illumina or Pacific Bioscience systems [13]. However, for those users who wish to generate new sequencing data sets with specific features, identification and generation of ground truth data sets is a laborious and cost-prohibitive endeavour. Moreover, it is extremely difficult to empirically determine the analytical consequences of different sample processing methods, experimental variability in library preparation and issues of sequencing bias in analysis [14].

Simulating NGS data provides an inexpensive alternative for assessing new algorithms in the context of sequencing data variation as noted [15]. With simulated datasets, one can

start refining analysis procedures *in silico*. Simulated NGS datasets can incorporate the variability associated with NGS sequence data including: sequencing coverage; number of libraries and insert size; base error rates; tool parameters at the data analysis level. For in silico NGS data, a large number of SV characteristics can be readily designed including the number, the category, the size, the breakpoint sequence, the variant fraction and the haplotype for any given locus. As a result, investigators can use this simulated data to assess the potential performance and make the trade-off between analysis cost and sensitivity before even carrying out the experiment.

Several programs generate NGS read sets to simulate metagenomics or single nucleotide polymorphisms are available [16-22]. Only recently have we seen the development and release of structural variant simulators. An early example is RSVSim [23], an R package which amends template sequence files with structural variant changes. However, this program requires an interactive R session and as a result, does not support batch processing. SCNVSim [24] improves upon RSVSim by providing a command line interface. It simulates somatic copy number variants given a number of desired SV events and/or contigs. Nonetheless, both SCNVSim and RSVSim produce very limited variant-containing contig files (FASTA), which require external steps to simulate sequence reads (FASTQ) and output resulting alignments (BAM). VarSim [14] improves upon RSVSim and SCNVSim with integrated read simulation using read simulators such as ART [25]. Instead of using a template sequence file, BAMSurgeon [26] patches an existing alignment file to embed structural variants. However, this application requires a high depth of coverage in the existing BAM file to successfully assemble a local contig for sequence patching. Moreover, the resulting structural variant may not have the exact breakpoints for the intended simulation. Overall, none of the listed tools provide a straightforward, joint control of an individual variant, including its exact breakpoints, ploidy and locus-specific allelic fraction.

These more complex features are particularly useful in simulating the clonal expansion of somatic structural variants as seen in tumors.

As a solution to the limitations of current structural variant simulators, we designed and implemented SVEngine, a full-featured simulation program suite. SVEngine is capable of generating short sequence read sets, such as produced by an Illumina system, for thousands of spike-in variants that cover different types, sizes, haplotypes and allelic fractions. Our application produces these simulated NGS data sets in a fraction of the time of other similar tools. SVEngine's flexibility for accepting different formats enables a user to generate whole genome or targeted sequencing data mimicking germ-line, somatic and complex clonal structured genomes with ease. It offers a high degree of allelic control through its parallelized divide-and-conquer planning scheme. In the simplest mode, users only need to provide the template (reference) sequences and a desired meta-distribution of type, size and variant frequency to receive a full set of resulting FASTA, FASTQ and BAM outputs along with the ground truth BED file.

SVEngine features and simulation performance

We compare the available features of SVEngine with other simulators that include RSVsim [23], SCNVsim [24], VarSim [14] and BAMsurgeon [26], as shown in **Table 1**. SVEngine and the other tools can simulate common types of copy number events, *e.g.* deletions and tandem duplications. All simulators except SCNVsim simulate copy number neutral events, including insertions, inversions, and translocations. SVEngine improves the simulation of more complex SV events – it incorporates a variety of additional structural variant types originating from a combination of changes, such as inverted translocations, inverted duplications, duplicated translocation, and foreign sequence insertions. Users directly specify these events while preparing their input parameters – this process is more

streamlined compared to other tools. For example, viral genome sequence insertion, which is a hallmark of the genomes of infected cells as seen in viral diseases and cancers [27], is easily achieved with SVEngine, but not available with other simulation software except for BAMSurgeon.

In terms of input/output flexibility and ease of use, SVEngine provides automates template sequence modification, read simulation and read mapping steps. These features are not found in other simulators of SV events. Also, SVEngine is the only tool which outputs a full set of simulation results in standard formats, including altered contig sequence (FASTA), simulated short reads (FASTQ) and alignment (BAM) files **(Figure 1).** At the input step, all tools take in template sequences in FASTA format as the starting material, while BAMsurgeon additionally requires a pre-existing alignment file in BAM format as input. Overall, read coverage of this BAM file has to be large (typically >30x), to successfully assemble local contigs. Such requirements preclude the use of BAMSurgeon in applications generating low coverage and consequently limit its users to mimicking conditions based on available high-coverage BAMs. The VarSim tool needs structural variant prototypes from DGV [28] making it only applicable to the human genome. At the output step, RSVsim and SCNVSim provide modified sequence contigs in FASTA files. BAMsurgeon mainly outputs modified alignment in BAM files. The associated contigs need to be extracted from log files. VarSim provides both contigs containing a variant and simulated short reads, but it still requires additional user effort to generate alignment files.

With regard to precise and versatile control of individual variants, SVEngine enables one to easily specify variant type, size, exact breakpoint, ploidy and allelic fraction for individual loci. Additionally, SVEngine simulates a full spectrum of germline, somatic and clonal structural variations by the specified meta-distribution. In comparison, RSVSim does not support loci-level control, as it only patches template sequence on demand. With SCNVsim and VarSim,

one only controls a meta-distribution of structural variants, such as the total number for each variant type, minimum and maximum variant size. SCNVsim allows the specification of ploidy, number and type of clones but does not have the capability to specify exact breakpoints. VarSim randomly resamples breakpoint and other variant information from a DGV database dump. Only BAMsurgeon and SVEngine support locus-specific variant fractions, *i.e.* allowing different allele fractions for individual variants. Moreover, only SVEngine supports locus-specific ploidy, *i.e.* allowing a different ploidy state for individual variants. Both BAMsurgeon and SVEngine also support exact breakpoints for individual variants. However, in practice, the actual breakpoints generated by BAMsurgeon may differ from input, as a result of improvised local contig assembly. Another unique feature of SVEngine is the ability to specify multiple sequencing libraries, which can each have different insert size mean and standard deviation, intended coverage depth, and read length.

In addition to the features listed in **Table 1**, SVEngine allows users to designate some regions while avoiding others. Examples of such applications include simulating exome or targeted sequencing data sets. This feature enables one to avoid complex regions such as telomeres and centromeres. SVEngine also features parallelized simulation by dividing genome into pieces, embedding variants into each piece and then stitching them together. Therefore, its performance can be boosted using the multi-core processors.

Table 2 lists the runtime on a test set of 15,000 SV events into a 30x coverage whole genome sequencing simulation, including 2,500 that consist of deletions, tandem duplications, inversions, translocations and domestic or foreign sequence insertions. In multi-processor mode, SVEngine has the shortest runtime in all three levels of simulation, i.e. obtaining altered contigs, simulated reads and alignments in FASTA, FASTQ and BAM formats in less than 10 min, 20 mins and 3 hours, respectively. Overall, SVEngine is 1x, 15x and 48x times faster than the single-process SVEngine run. The performance scales almost

linearly with the added CPU power in generating the alignment output, because the read mapping time cost dominates other time costs including data serializing time. Finally, even the single-process SVEngine (SVE-single) is more efficient than its other counterparts. For example, it took only 10 mins for SVE-single to generate all altered contigs when RSVSim and SCNVSim took several hours. SVE-single required half the time BAMSurgeon needs to generate all read alignments. All run time were measured on a computer server with four Intel® Xeon E7-4850 CPUs (with 16 cores each CPU) and with 256 GB shared RAM.

Simulating cancer genome evolution

SVEngine provides a high degree of control over SV events with variable allelic fractions – this feature enables one to simulate heterogeneous cancer genomes undergoing a phylogeny tree-structured clonal evolutionary process. As a demonstration, we present an example simulated with SVEngine **(Figure 2)**. To simplify the description of the phylogenetic process of cancer evolution, we use a binary tree representation of phylogeny. This binary tree is easily converted to a typical phylogeny tree by merging all nodes of identical cell subpopulations.

One example is a binary tree shown in **Figure 2A**, where each of the five ($m = 5$) internal tree nodes denotes a bifurcation event when part of the parental cell population is gaining an additional mutation ($V_j, j = 1 \dots m$). The root node represents the lowest common ancestor cells of all subpopulations of cancer cells. These are typically normal cells that carry a genome that matches a germline genome, subsequently from which somatic genetic alterations accrue as part of cancer development. The root cell populations are split by the next immediate event, i.e., gaining the mutation V_1, resulting in two daughter cell populations depending on a cell's status of carrying V_1 or not, as represented by its two daughter cellular node. We denote the conditional cell fraction of gaining V_1 as $f(V_1)$, which is 50% or 0.5 in

this case, and is denoted at the root. The mutational process goes on for subsequent internal nodes and until all variants (a total of five in this example) are represented by their bifurcation internal node. The resulting binary tree has six ($n = 6$) leaf nodes ($C_i, i = 1 \dots n$), which represent all possible somatic genotypes of the terminal cell subpopulations.

As we can see, any terminal somatic genotype is completely determined by following the mutational path from the root down to a leaf node. We use a tertiary vector $C_i = (c_{i,1} \dots c_{i,m})$ to indicate such path, where

$$c_{i,j} = \begin{cases} 0, & \text{if } V_j \text{ is not in the mutation path to } C_i \\ 1, & \text{if } V_j \text{ is in the mutation path to } C_i \text{ but } C_i \text{ doesn't carry } V_j \\ 2, & \text{if } V_j \text{ is in the mutation path to } C_i \text{ and } C_i \text{ does carry } V_j \end{cases}$$

In addition, we define the conditional frequencies $f(V_i)$, which is the fraction of cells derived from a parent population carrying event V_i as: $f(V_i) = \frac{\#\text{ Child cells Gains } V_i}{\#\text{ Parent cells at the verge of gaining } V_i}$.

Therefore, the final population frequency $F(C_i)$ of cell subpopulations C_i is expressed as:

$$F(C_i) = \prod_{j:c_{i,j}>0} [(c_{i,j} - 1) * f(V_i) + (2 - c_{i,j})(1 - f(V_i))] \quad (1).$$

With $I(.)$ as the indicator function, the concurrent proportion $F(V_j)$ of all extant cells is simply the marginal sum of all cells carrying V_j:

$$F(V_j) = \sum_{i=1}^{n} I(c_{i,j} = 2) F(C_i) \quad (2).$$

Figure 2B shows the derivation of the above quantities for the example binary tree. The sequence of events ensures a partial order that the mutant allele frequency is always higher for events occurring upstream, as compared to events occurring downstream on the same lineage. It is possible that terminal genotypes may not all coexist in extant populations. The proposed binary tree representation accommodates a deceased population by having zero proportion for such leaf node. SVEngine allows user to input a binary tree with relevant bifurcation fractions to structure the variant fractions that fall along the line of the evolutionary tree. For designating this feature, the input to SVEngine is in standard NEWICK format – a widely accepted format using parentheses to encode nested tree structures [29]. Each internal node is labelled by the population splitting variant and weighted by the conditional splitting fraction. Each leaf node is labelled by associated terminal genotype and weighted by the subpopulation fraction as an optional feature. For instance, the NEWICK string for the example binary tree is: ((C1, C2) V5: 0.8, ((C3, C4) V4:0.8, (C5, C6) V3: 0.9) V2:0.6) V1:0.5.

Figure 2C shows the IGV browser view of SVEngine simulated BAM alignments of five equal-size deletions following the mutational process as represented by the example binary tree. The read depth shows the difference of allelic fractions corresponding to the computed final variant fractions based on the tree. We display an example of monoclonal cancer evolution, assuming that all cellular subpopulations start from a set of common ancestor cells (as denoted by the root node in the tree). In addition, simulations of multiclonal evolution are also possible with SVEngine. For example, one simply assigns an empty event to V_1 and then sets the conditional fractions of the two child events V_1 and V_5 to 100% to simulate a two-clonal origin evolution. With SVEngine's high efficiency, the simulation is easily scaled to tens of thousands of variants with a tree having a more complex structure.

Simulating the multitude of structural variations

Current structural variation detection methods mostly rely on detecting altered read mapping features to identify structure changes [30]. The most important such features are read depth/coverage, read pair insert size, single ended read pairs (hanging reads), soft-clipped reads and split reads (clip/split reads). It is essential for structural variant simulators to correctly produce such feature changes corresponding to the causal event. In **Figure 3**, we comprehensively illustrate the expected changes in mapping that result from different types of structural variants.

In the scenario of a deletion **(Figure 3, first row)**, all the mapping features, such as coverage, insert size, hanging read and soft-clip/split read are expected to change, as illustrated in the *Coverage*, *InsertSize, HangingRead* and *ClipSplitRead* columns, respectively. First, there is a reduction of read coverage over the deleted region because no reads are present. Second, for those read pairs that are mapped straddling the breakpoints, the insert size is expected to increase as inferred by alignment to the reference. This extended insert size is possible because the deleted region is not present in the real DNA molecules where these read pairs originating from. Third, a fraction of read pairs aligning to the left of the left breakpoint lack a mapped mate read – this generates a right mate hanging read pair. This phenomenon is also called a right hang. This phenomenon occurs because the left breakpoint has interrupted the mate mapping by reducing similarity between the read and the reference. Due to symmetry, left mate hanging read pairs (left hang) form to the right of the deletion. Finally, when the breakpoint interruption in the mate is limited to the end of the read, it is possible that the mate read can still partially map. The non-contiguous part of the mate is either clipped or, if it is long enough, mapped to near the other end of the deletion. Such resulting read pairs are what we refer to as left or right soft-clipped (or split mapped) reads depending on which side of the reads were split or clipped. These read pairs are expected to map right next to both breakpoints with the clipping (or splitting site) aligned to the exact break point location, as shown.

For an insertion **(Figure 3, second row)**, the most noticeable change is the clustering of both the right and left hanging read pairs centering over the breakpoint. One observes a similar clustering for the left and right clip/split reads. As shown in **Figure 3**, an insertion exhibits fewer changes than other types of structural variants, and so insertions are generally the most difficult to detect. In the scenario of a tandem duplication **(Figure 3, third row)**, the read coverage is expected to increase within the duplicated region. The insert size of reads mapping to the left of the right breakpoint is expected to decrease, or even produce a negative value based on the duplication's position in the chromosome. This is the case because the mate is likely to have the same sequence as the segment preceding the read. Then, when the mate is mapped upstream of the current read, it causes a reversal of normal read strand order and introduces a negative insert size in the read mapping. By the same reasoning, the right hanging, clipped and split reads are clustered upstream next to the right breakpoint. Similarly, the left hanging, clipped and split reads are clustered downstream next to the left breakpoint, making the tandem duplication almost a mirror image of deletion.

In the scenario of an inversion **(Figure 3, fourth row)**, the coverage shows almost no change. The insert size near the left breakpoint is similar to the deletion scenario, which has an increase. This occurs as a result of the mate from the reverse complement of the other end of the inverted segment – this scenario creates an inflated insert estimate and an abnormal forward-forward strand read pair. Similarly, the insert size near the right breakpoint is decreased and forms an abnormal reverse-reverse strand read pair. When these abnormal pairs are interrupted by the breakpoints, it creates corresponding hanging read and clipped/split read clusters around both breakpoints. Citing another example, a chromosomal translocation is simply a combination of features at the region deleted by the translocation and insertion features at the region inserted.

Simulation benchmark with NA12878

We additionally validated SVEngine's simulated data by applying popular structural variant callers and a tumor heterogeneity estimating tool to SVEngine generated WGS data and benchmarked their performance with SVEngine's input ground truth. For our initial benchmark, we simulated WGS data for a well-studied individual NA12878 based on her known variants [31]. We applied the commonly used SV callers: Lumpy [2] and Manta [10] and computed performance metrics such true positive rate (TPR or sensitivity) and false discovery rate (FDR) for the callers at different simulated coverage. In total, we simulated 20,759 structural variants for NA12878 with exact genotype and breakpoint information. The set included 19,034 deletions, 1,150 duplications, 328 inversions and 247 insertions. These insertions included 91 LINE1, 58 ALU and 9 SVA mobile element insertions, for which we determined the exact inserted sequence using RepBase [32].

As shown in **Figure 5** (**A**) and (**B**), both SV callers performed well on the SVEngine simulated data. For Manta, the overall TPR (i.e. true positive rate or sensitivity) ranges from 60% at 10x of coverage to 98% at 100x. For Lumpy, the TPR is 40% at 10x of coverage and raise to 88% at 100x. There seems to be a critical coverage value at around 25x, above which both callers can reach >80% sensitivity. This is in agreement with the widely accepted empirical coverage choice at 30x for WGS. The overall FDR (i.e. false discovery rate) for Manta was consistently within the 2%-3% range for average coverage ranging from 10x to 100x. The FDR for Lumpy was considerably higher from <1% at 10x to 23% at 100x. This is counterintuitive since we generally assume that higher coverage leads to improved performance and not the other way around. Why is this so for Lumpy? The deviation of the two tools in FDR may result from their different calling strategy. Manta likely has a dynamic coverage-based threshold. Lumpy's default parameter (as provided in its online manual) is likely tuned for <50x coverage for optimal performance. For higher coverage data, Lumpy may need to be calibrated and this can be done using SVEngine's simulated data. As SV

typing is still challenging for SV callers, to compute these overall TPR and FDR, we required only adjacent match of predicted breakpoint and the ground truth.

If one considers the SV type (see **Supplementary Table S1**), deletions are the least challenging SV type for all callers. Manta had a TPR ranges from 60% to 99% as the coverage increased from 10x to 100x. Its FDR was consistently low at <1.2%. The same was true for Lumpy, which had a TPR increase from 42% to 88% with a consistently low FDR at <2%. Calling inversions was also well achieved by the callers. Manta and Lumpy had a TPR from 83% to 100% and 66% to 97.3%, respectively, with increasing coverage. Both maintained a FDR that was approximately zero. Their performance diverges when it came to calling duplications and insertions. Manta maintained a good performance at calling duplications, with TPR increasing from 47.5% to 90.6% with coverage and the FDR maintained close to zero. Its performance with insertion was less impressive, as the TPR increased from 0% to 41% with coverage with considerable FDR in the range of 30-40%. On the other hand, Lumpy did not correctly type any duplications and insertions and breakpoints of these SV types were more likely to be classified as unknown. Lumpy had a significantly increasing number of untyped calls with higher coverage. Between 30% to 50% of such calls were validated breakpoints of duplications and insertions. Generally, the SV callers performed well on calling and typing deletions, inversions and duplications with SVEngine simulated data. Manta had the best overall performance. Calling and typing insertions was the most challenging task for SV analysis. SVEngine simulation of insertions will aid the further development of insertion callers.

Simulation benchmark with tumor heterogeneity

To validate SVEngine's correctness in simulating various mutant allele frequency, we simulated a series of WGS data representing two-population mixtures of tumor and normal

genomes at a chosen level of tumor purity. We derived copy number segment files from the SVEngine simulated data set and ran the tumor heterogeneity caller THetA2 [33] to infer the mixing subpopulation frequency.

We compared the THetA2 estimates to the input ground truth purity to SVEngine in **Figure 5** (**C**) and **Supplementary Table S2**. The THetA2 estimated purities are (0.165, 0.234, 0.466, 0.728, 0.844) for input ground truth purities at (10%, 25%, 50%, 75%, 90%) respectively. The root mean square deviation (RMSD) is 0.043 and the Pearson's correlation coefficient is 0.996 (i.e. $R^2 > 0.99$). The high correlation and small error measure indicate SVEngine's correct simulation of tumor and normal mixture WGS data. The copy number segments derived from such data are suitable input to standard tumor heterogeneity tools such as THetA2 for reliably estimating tumor purities, even for simulating extreme cases where the tumor purity is as low as 10%.

CONCLUSION

We have developed and released SVEngine, a structural variant simulator, available as an open source program. It simulates next generation sequencing data that has embedded structural variations as well as an assortment of complex sequence features. SVEngine simulates and outputs mutated sequence contigs (FASTA), sequence reads (FASTQ) and/or alignments (BAM) files with desired variants, along with BED files containing ground truth. SVEngine's flexible design enables one to specify size, position, and heterogeneity for deletion, insertion, duplication, inversion and translocation variants. SVEngine's additional features include simulating sequencing libraries having multiple different molecular parameters, and targeted sequencing data sets. SVEngine is highly parallelized for rapid and high throughput execution.

We showed the versatility and efficiency of SVEngine by comparison of features and runtime versus other available simulators. We demonstrated the utility of SVEngine in an example mimicking the phylogeny in cancer clonal evolution, by simulating the associated variant allelic frequency. We validated the accuracy of SVEngine simulations by examining expected sequence mapping features such as coverage change, read clipping, insert size shift and neighbouring hanging read pairs for representative variant types. SVEngine is implemented as a standard Python package and is freely available for academic use at: https://bitbucket.org/charade/svengine.

The analysis of structural variants is an important part of genomics research. Improvements in the field also come from a growing set of available technologies, *e.g.* long read technologies such as the single-molecule, real-time (SMRT) sequencing by Pacific Biosciences [34] and the nanopore sequencing by Oxford Nanopore Technologies [35], or synthetic long read technologies (SLR) such as the Chromium droplet-based library preparations by 10X Genomics [36-38]. As the empirical data from these technologies accumulate, platform specific read simulators like PBSIM [39] and NanoSim [40] will become increasingly available. Although the implementation is non-trivial, the design of SVEngine is fully compatible with alternative read simulators. Going forward, we will work with the community to expand SVEngine with more powerful features, such as multi-platform simulation and co-phased SNP simulation.

METHODS

Simulation software and pipeline

SVEngine was developed as a standard Python package with a C extension. SVEngine provides two Python executables and one C command line executable: *mutforge*, *tree2var* and *xwgsim,* respectively. The *mutforge* command implements a parallelized algorithm that divides the template genome into blocks of contigs, spikes structural variants into the contigs, samples short reads from the altered contigs, and finally merges the short-read sets back into one file and performs the alignment. The *tree2var* command implements a procedure that determines variant fractions from an input phylogeny tree based on **Equations (1)** and **(2)** and a depth first search graph algorithm, and then substitutes these allele fractions in an input VAR file. The *xwgsim* command implements a modification to *wgsim*, which reduces the read sampling rate by 50% for the overlapping regions between contigs (i.e. ligation regions). The overlaps were designed so as to allow for the proper merging of contig-wise read sets. *xwgsim* only interacts with *mutforge* thus is mostly transparent to a user.

As shown in **Figure 1**, the required inputs to *mutforge* are three-fold: 1) a template haploid sequence file(s) in FASTA format. This can be a standard human genome reference, or any other reference genome sequence. 2) A VAR file or a META file for specifying structural variants (distributions). These are tab delimited files with columns defined in SVEngine's manual. The VAR format is intended for specifying exact information for individual variants, which includes variant id, parent id (if part of a complex event such a deletion occurring due to a translocation), fraction, ploidy, chromosome, starting position, and the sequence length to be deleted and/or the sequence content to be inserted. Alternatively, the META format is intended for higher-level control, allowing one to specify a desired meta distribution of variants, including variant type, and total number of events, size, allele fraction, and ploidy distributions per type.

One can specify where and how to insert the sample sequence in the case of foreign DNA insertion. For example, a user can readily design 100 deletions of size ranging from 100bp to 10kbp, of a uniform distribution of allelic fraction and a fair Bernoulli distribution of homo- and heterozygosity in one line of text in the META file. 3) The PAR file is used to model an experimental design, including insert size, read length and coverage, as well as additional options for *xwgsim*. The file can be used to specify multiple libraries with different mean insert size and standard deviation. One can use such normal mixtures to approximate irregular libraries of multiple modes and asymmetric tails. The *xwgsim* command also provides random embedding of SNVs and indels if desired. In the SVEngine's Wiki page, we supply example VAR, META and PAR files with detailed annotation to facilitate their usage.

Once all inputs are provided, the SVEngine master process divides the template genome into blocks and serializes spike-in tasks to parallel worker processes. The worker process patches its assigned contig and if read pairs were required, it also calls *xwgsim* to simulate read pairs. The read pair subsets are then collected by the master process and merged, and if alignments were required, it also calls *bwa-mem* and *samtools* to map the reads to the reference.

The output of SVEngine has three levels. At the first level (contig), only two files would be generated: one is a FASTA file containing all the altered contigs, the other is the ground truth of spiked-in variants in a BED3 format file with the additional columns following the VAR format as in the input. At the second level (read pair), SVEngine additionally outputs the read 1 and read 2 of the simulated read pairs in two FASTQ files. Finally, at the third level (alignment), SVEngine provides the read alignment output to the given reference in a BAM

file format. The runtime of SVEngine increases with the specified output level, as additional processing time will be required. Table 2 can be used as a reference for runtime estimates for different output levels.

The *tree2var* command simulates a clonal evolution scenario, which requires an additional tree input file (NEWICK). *tree2var* also takes a VAR file, which can be generated from *mutforge* with a META file input and in the dryrun mode. The user must insure that the identifier of the tree's internal nodes and the variant match each other, as this is used to identify and replace allele fraction with the value computed from tree phylogeny. The *tree2var* outputs a new VAR file which contains the rewritten allele fraction fields that reflects the clonal structure described by the user tree. For intuitive diagnostics, *tree2var* also outputs a ASCII text based plot of the parsed input tree. SVEngine's tree parsing interacts with DendroPy [29], which allows further functionality such as random tree simulations and many tree statistics. The output VAR file from *tree2var* then becomes the input to *mutforge* for actual read simulation.

A parallel simulation framework

SVEngine's major improvements to existing structural variant simulation tools involve one's ability to alter the allelic fraction, control of haplotypes and highly efficient parallelized simulation. These improvements were achieved through the core algorithm as illustrated in **Figure 4**. In general, we used a divide-and-conquer approach intertwined with multi-process execution: First, the SVEngine master process lays out a genome grid for simulation. For any input haploid sequence, the entire genome is partitioned into N equal size non-overlapping blocks: $B_1, B_2, \dots, B_N$, where $B_k = \left[\frac{G(k-1)}{N} + 1, \frac{Gk}{N}\right]$. The planned block size $\frac{G}{N}$ (i.e. plan size in the manual) can be chosen at the input, where G is the entire genome length. Ligation regions of length l (i.e. ligation size in the manual) are also defined, which consists

of symmetric touching border regions of equal size adjacent blocks: $L_1, L_2, \ldots, L_{N-1}$, where $L_k = [\frac{Gk}{N} - \frac{l}{2} + 1, \frac{Gk}{N} + \frac{l}{2}]$. These serve as buffer regions that enable the SVEngine to ligate block sequence-based simulations back together. The block generating procedure is similar for multi-chromosome genomes except blocks representing chromosome ends might be shorter than the standard block size.

Second, the SVEngine master process coordinates all of the tasks. In one task, a structural variant is embedded into the adjacent sequence – this is done by assigning a sequence of blocks that it impacts. All the variant's control information is attached to the task as well. In the figure, the first variant, a 50% deletion SV_i was assigned blocks $B_{i_1}, B_{i_1+1}, \ldots, B_{i_n}$ and the next variant, a 100% deletion SV_j was assigned blocks $B_{j_1}, B_{j_1+1}, \ldots, B_{j_n}$. Depending on its size, a variant can take anywhere from one block, to as many blocks as needed. The genomic region which is not altered, between adjacent variants, say SV_i and SV_j, also becomes a task. This is assigned to the sequence blocks complementary to the blocks taken by SV_i and SV_j and with a no-op instruction attached. If necessary, no-op tasks with large block sequences are further broken down to no-op tasks with size-capped block sequences to improve efficiency of parallelization. The size cap is defined by the trunk size option as explained in the manual.

Third, the SVEngine master process dispatches all the tasks to an auto revolving worker process pool and then waits for all the workers to finish. Each worker process, when assigned a new task, loads the haploid sequence defined by the task's block sequence plus left and right ligation regions. For example, a worker would load sequence from $\left[\frac{Gi_1}{N} - \frac{l}{2} + 1, \frac{Gi_n}{N} + \frac{l}{2}\right]$ for SV_i as the original contig, or $\left[\frac{Gj_1}{N} - \frac{l}{2} + 1, \frac{Gj_n}{N} + \frac{l}{2}\right]$ for SV_j, or $\left[\frac{G(i_n+1)}{N} - \frac{l}{2} + 1, \frac{G(j_1-1)}{N} + \frac{l}{2}\right]$ for the no-op task in between them. The original contig is then operated on for

deletion, insertion, or other alternations to form the altered contig. If no-op the original contig is unaffected. The worker then calls *xwgsim* to simulate the proper numbers of read pairs from the original and altered contigs according to the specified frequency and resulting contig sizes. The *xwgsim* step also takes care of attenuated sampling (at half the normal rate) within the designated ligation regions as the worker provides the ligation size l in its arguments. In addition, *xwgsim* adds a procedure to the popular NGS simulator *wgsim* [41] to correctly adjust coverage for a ligation region. Briefly, we define the ligation region as a segment of haploid sequence where two adjacent contigs to be simulated overlaps. The ligation region is employed to ensure proper and continuous transition from simulating reads from the first contig to the second. That also means a ligation region would be simulated twice – once along with the first contig, and then along with the second contig. To compensate for potential double coverage in a ligation region, we implemented an adjusted read generation procedure in *xwgsim*, which only simulates 50% of the intended read coverage within the region for one contig. Such patterns of expected read pair coverage from the SV_i, SV_j and no-op tasks are illustrated in **Figure 4**.

Fourth, when the worker processes are completed, the master process collects all simulated read pairs from all tasks and concatenates them into two final files, one for read 1 and the other for read 2. Also, it collects all original and altered contigs and concatenate them into one final sequence file. Finally, it performs read pair alignment to the reference genome using *bwa-mem* and *samtools*. This is last step, although sequential in SVEngine, is already thread parallelized by other required programs such as the *bwa* and *samtools* tools [22, 42]. Patterns of expected read pair coverages after merging the SV_i, SV_j and no-op tasks are also illustrated in **Figure 4**. The described algorithm assumes one haploid for simplicity. For multi-ploidy, each haploid is handled in a similar way by the worker process except that the variant’s haplotype status is also taken into consideration. Overall, this SVEngine’s core

algorithm is very efficient as demonstrated by the runtime comparison, is very versatile and accurate as demonstrated by multiple example applications described in this paper.

Notable simulator features

To comprehensively evaluate structural variant callers, one may need a wide spectrum and large number of SV events. This range is more easily specified by distributions of variants rather than individual variants. SVEngine supports variant distributions as specified in the META format. The expansion of distributions to actual variants takes place in the master process before any spike-in. The distributions are expanded on a target genome sequentially by randomly pick the next event's start position from regions that can accommodate it. Afterwards, it removes the impact region from the remaining available regions and so on. Once all distributions are expanded, the master process returns a list of variant fulfils user's specification and outputs them into a VAR file. The user can choose to run SVEngine in dryrun mode to stop the execution at this point and inspect the resulted variants. The user also has the option to continue the simulation to the end, which is equivalent to input the output VAR file into SVEngine for simulation in the next step.

To increase the sensitivity of SV detection, researchers may prepare multiple sequencing libraries with different molecular parameters for analysis. For example, different insert sizes enable the detection of a wider spectrum detection of SVs [43]. Longer sequence read length can boost the performance of some callers that employ remapping strategies [44]. A unique feature of SVEngine is its ability to simulate NGS data modelling multiple libraries with different mean insert size and standard deviation, coverage and read lengths. The feature is implemented within the worker process. When using a multi library task, SVEngine will call *xwgsim* multiple times to generate read pairs in accordance with the library specification.

SVEngine provides simulation data that target or masks specific genomic regions. This feature emulates targeted sequencing applications, such as exome sequencing and gene panel sequence data. It can be used to exclude problematic regions such as gaps, telomere and centromere regions of the reference template. One only needs to provide standard BED format files to SVEngine listing the regions to be masked or targeted by the simulated sequencing.

Databases like DGV and much other literature provide a list of known population variants. Tools like RepeatMasker (http://repeatmasker.org) provide extensive lists of known regions of human repeats and/or homolog sequences, with enrichment of structural variant breakpoints. Although not provided in our examples due to their varied formats, in principle, these population and repeats-mediated variants can be downloaded in general tab delimited formats, such as BED or VCF files. Subsequently, these annotation formats are easily converted into an SVEngine VAR format input file using text processing utilities such as *awk* and *sed*. Using a VAR file generated in this way, SVEngine can easily embed these variants into simulation data.

Simulation benchmark with NA12878

To validate SVEngine's correctness in simulating various structural variants, we simulated WGS data for a well-studied individual NA12878 based on her known variants as published by the 1000 Genomes Project (1KG) [31], ran commonly used SV callers: Lumpy [2] and Manta [10] and computed performance metrics such true positive rate (TPR or sensitivity) and false discovery rate (FDR) for the callers as an indication of our simulation correctness.

In detail, we downloaded 1KG's NA12878 final call set from its ftp site (ftp://ftp.1000genomes.ebi.ac.uk/vol1/ftp/phase3). We also downloaded human mobile element sequences form RepBase [32] (version 23.02). After cleaning up inconsistent variants registered by multiple callers and those were mobile elements insertion without available RepBase sequences, we arrived at 20759 structural variants for NA12878 with exact genotype and breakpoint information. The set included 19034 deletions, 1150 duplications, 328 inversions and 247 insertions. These insertions included 91 LINE1, 58 ALU and 9 SVA mobile element insertions, the inserted sequence of which we were able to determine from the RepBase.

We encoded the information of these variants, such as SV type, genotype, breakpoint and inserted sequences into a VAR format suitable for input to SVEngine. We specified the sequencing library with a mean insert size of 300 and the sequencing run with 2 x 150bp pair-end read and ran SVEngine to generate a series of WGS data in BAM files with coverage of 10x, 25x, 50x, 75x and 100x. The latest version of Lumpy and Manta were downloaded from their GitHub site and installed. We applied them on the SVEngine generated WGS data series with their default parameters. The resulting SV calls were parsed into BED files and compared with ground truth VAR file using BEDTOOLS. We consider a true positive hit if a call's breakpoint is within 20 base pairs of ground truth. We compared caller's performance both with and without enforcing correct SV typing. The performance metrics true positive rate (TPR) or sensitivity and false discovery rate (FDR) were calculated as following:

$$True\ Positive\ Rate\ (TPR) = \frac{\#\ of\ True\ Positives}{\#\ of\ Ground\ Truth}\ \%$$

and

$$False\ Discovery\ Rate\ (FDR) = \frac{\#\ of\ Calls - \#\ of\ True\ Positives}{\#\ of\ Calls}\ \%.$$

Simulation benchmark with tumor heterogeneity

To further validate SVEngine's correctness in simulating various mutant allele frequency, we simulated a series of WGS data representing different mixtures of tumor and normal genomes, ran the tumor heterogeneity caller THetA2 [33] on the simulated data derived segment file and compared the THetA2 estimates to our ground truth purities.

In detail, we downloaded and installed the latest version of THetA2 from its GitHub site. We simulated a two-subpopulation scenario, where one tumor cell population and one normal cell population were mixed. We used the example segmental intervals provided by THetA2 as the ground truth copy number status of the tumor cell population and the human reference genome for the normal cell population. The copy number file has 84 segments, including 45 neutral, 26 losses and 13 gains. We accordingly encoded these copy number variants into a series of VAR files with allelic frequencies: 10%, 25%, 50%, 75% and 90%. We used SVEngine to generate WGS data BAM files based on the series of VAR files, with mean insert size 300bp, 2 x 150bp pair-end read and 100x coverage. We used *featureCounts* [45] to compute probe segment file suitable for input to THetA2 and ran THetA2 with default parameters to estimate the tumor heterogeneity. We regressed the THetA2 estimated purity over SVEngine ground truth purity to find the R^2 statistic with the lm function in R.

Availability of supporting source code and requirements

Project name: SVEngine

Project home page: https://bitbucket.org/charade/svengine

Operating system: Linux/Unix

Programming language: standard Python package with a C extension

Other requirements: GNU C Compiler or similar

License: BSD3

Research Resource Identifier (RRID): SVengine, SCR_016235

Data availability

In silico data sets are available via Bitbucket [46]. An archival copy of the Bitbucket repository is also available via the *GigaScience* database GigaDB [47].

Abbreviations

- SV: Structural Variation/Variant
- NGS: Next Generation Sequencing

Ethics approved and consent to participate

Not applicable. No human subjects or data. No animal subjects or data.

Consent for publication

Not applicable.

Competing interests

The authors declare that they have no competing interests.

Funding

LCX, NRZ and HPJ were supported by National Institute of Health (NIH) R01HG006137. NA was supported by National Cancer Institute's (NCI) Cancer Target Discovery and

Development (CTDD) Consortium (U01CA17629901), NCI K99 CA215256 and the Don and Ruth Seiler Fund. HPJ was also supported by NIH P01 CA91955. HJL and HPJ were supported by NIH U01CA15192001. DMA and CL were supported by the National Natural Science Foundation of China (61370131). Other support for HPJ came from the Gastric Cancer Foundation and the Research Scholar Grant, RSG-13-297-01-TBG from the American Cancer Society.

Author contributions

LCX, NRZ and HPJ designed the study. LCX developed the algorithm, wrote the program and tested the software. HJL and NA provided feedback on the software design and testing. LCX and DMA generated and analyzed the data with assistance from CL. LCX and HPJ wrote the manuscript. All authors approved the manuscript.

Acknowledgements

We thank John Bell, Sue Grimes and Erik Hopmans at Stanford University and Yuchao Jiang at University of Pennsylvania for helpful discussions.

REFERENCES

1. Alkan C, Coe BP, Eichler EE: **Genome structural variation discovery and genotyping**. *Nat Rev Genet* 2011, **12**(5):363-376.
2. Layer RM, Chiang C, Quinlan AR, Hall IM: **LUMPY: a probabilistic framework for structural variant discovery**. *Genome Biol* 2014, **15**(6):R84.
3. Chen K, Wallis JW, McLellan MD, Larson DE, Kalicki JM, Pohl CS, McGrath SD, Wendl MC, Zhang Q, Locke DP *et al*: **BreakDancer: an algorithm for high-resolution mapping of genomic structural variation**. *Nat Methods* 2009, **6**(9):677-681.
4. Rausch T, Zichner T, Schlattl A, Stutz AM, Benes V, Korbel JO: **DELLY: structural variant discovery by integrated paired-end and split-read analysis**. *Bioinformatics* 2012, **28**(18):i333-i339.
5. Abyzov A, Urban AE, Snyder M, Gerstein M: **CNVnator: an approach to discover, genotype, and characterize typical and atypical CNVs from family and population genome sequencing**. *Genome Res* 2011, **21**(6):974-984.
6. Ye K, Schulz MH, Long Q, Apweiler R, Ning Z: **Pindel: a pattern growth approach to detect break points of large deletions and medium sized insertions from paired-end short reads**. *Bioinformatics* 2009, **25**(21):2865-2871.
7. Xia LC, Sakshuwong S, Hopmans ES, Bell JM, Grimes SM, Siegmund DO, Ji HP, Zhang NR: **A genome-wide approach for detecting novel insertion-deletion variants of mid-range size**. *Nucleic Acids Res* 2016, **44**(15):e126.
8. McKenna A, Hanna M, Banks E, Sivachenko A, Cibulskis K, Kernytsky A, Garimella K, Altshuler D, Gabriel S, Daly M *et al*: **The Genome Analysis Toolkit: a MapReduce framework for analyzing next-generation DNA sequencing data**. *Genome Res* 2010, **20**(9):1297-1303.
9. Moncunill V, Gonzalez S, Bea S, Andrieux LO, Salaverria I, Royo C, Martinez L, Puiggros M, Segura-Wang M, Stutz AM *et al*: **Comprehensive characterization of complex structural variations in cancer by directly comparing genome sequence reads**. *Nat Biotechnol* 2014, **32**(11):1106-1112.
10. Chen X, Schulz-Trieglaff O, Shaw R, Barnes B, Schlesinger F, Kallberg M, Cox AJ, Kruglyak S, Saunders CT: **Manta: rapid detection of structural variants and indels for germline and cancer sequencing applications**. *Bioinformatics* 2016, **32**(8):1220-1222.
11. English AC, Salerno WJ, Hampton OA, Gonzaga-Jauregui C, Ambreth S, Ritter DI, Beck CR, Davis CF, Dahdouli M, Ma S *et al*: **Assessing structural variation in a personal genome-towards a human reference diploid genome**. *BMC Genomics* 2015, **16**:286.
12. Sims D, Sudbery I, Ilott NE, Heger A, Ponting CP: **Sequencing depth and coverage: key considerations in genomic analyses**. *Nat Rev Genet* 2014, **15**(2):121-132.
13. Zook JM, Chapman B, Wang J, Mittelman D, Hofmann O, Hide W, Salit M: **Integrating human sequence data sets provides a resource of benchmark SNP and indel genotype calls**. *Nat Biotechnol* 2014, **32**(3):246-251.
14. Mu JC, Mohiyuddin M, Li J, Bani Asadi N, Gerstein MB, Abyzov A, Wong WH, Lam HY: **VarSim: a high-fidelity simulation and validation framework for high-throughput genome sequencing with cancer applications**. *Bioinformatics* 2015, **31**(9):1469-1471.
15. Ugaz VM: **Introduction to next generation sequencing and genotyping issue**. *Electrophoresis* 2012, **33**(23):3395-3396.
16. Richter DC, Ott F, Auch AF, Schmid R, Huson DH: **MetaSim: a sequencing simulator for genomics and metagenomics**. *PLoS One* 2008, **3**(10):e3373.
17. Angly FE, Willner D, Rohwer F, Hugenholtz P, Tyson GW: **Grinder: a versatile amplicon and shotgun sequence simulator**. *Nucleic Acids Res* 2012, **40**(12):e94.
18. McElroy KE, Luciani F, Thomas T: **GemSIM: general, error-model based simulator of next-generation sequencing data**. *BMC Genomics* 2012, **13**:74.

19. Johnson S, Trost B, Long JR, Pittet V, Kusalik A: **A better sequence-read simulator program for metagenomics**. *BMC Bioinform* 2014, **15 Suppl 9**:S14.
20. Yuan X, Zhang J, Yang L: **IntSIM: An Integrated Simulator of Next-Generation Sequencing Data**. *IEEE transactions on bio-medical engineering* 2017, **64**(2):441-451.
21. Pattnaik S, Gupta S, Rao AA, Panda B: **SInC: an accurate and fast error-model based simulator for SNPs, Indels and CNVs coupled with a read generator for short-read sequence data**. *BMC bioinformatics* 2014, **15**:40.
22. Li H, Handsaker B, Wysoker A, Fennell T, Ruan J, Homer N, Marth G, Abecasis G, Durbin R, Genome Project Data Processing S: **The Sequence Alignment/Map format and SAMtools**. *Bioinformatics* 2009, **25**(16):2078-2079.
23. Bartenhagen C, Dugas M: **RSVSim: an R/Bioconductor package for the simulation of structural variations**. *Bioinformatics* 2013, **29**(13):1679-1681.
24. Qin M, Liu B, Conroy JM, Morrison CD, Hu Q, Cheng Y, Murakami M, Odunsi AO, Johnson CS, Wei L *et al*: **SCNVSim: somatic copy number variation and structure variation simulator**. *BMC Bioinform* 2015, **16**(1):66.
25. Huang W, Li L, Myers JR, Marth GT: **ART: a next-generation sequencing read simulator**. *Bioinformatics* 2012, **28**(4):593-594.
26. Ewing AD, Houlahan KE, Hu Y, Ellrott K, Caloian C, Yamaguchi TN, Bare JC, P'ng C, Waggott D, Sabelnykova VY *et al*: **Combining tumor genome simulation with crowdsourcing to benchmark somatic single-nucleotide-variant detection**. *Nat Methods* 2015, **12**(7):623-630.
27. Sadeyen JR, Tourne S, Shkreli M, Sizaret PY, Coursaget P: **Insertion of a foreign sequence on capsid surface loops of human papillomavirus type 16 virus-like particles reduces their capacity to induce neutralizing antibodies and delineates a conformational neutralizing epitope**. *Virology* 2003, **309**(1):32-40.
28. MacDonald JR, Ziman R, Yuen RK, Feuk L, Scherer SW: **The Database of Genomic Variants: a curated collection of structural variation in the human genome**. *Nucleic Acids Res* 2014, **42**(Database issue):D986-992.
29. Sukumaran J, Holder MT: **DendroPy: a Python library for phylogenetic computing**. *Bioinformatics* 2010, **26**(12):1569-1571.
30. Alkan C, Coe BP, Eichler EE: **Genome structural variation discovery and genotyping**. *Nat Rev Genet* 2011, **12**(5):363-376.
31. Sudmant PH, Rausch T, Gardner EJ, Handsaker RE, Abyzov A, Huddleston J, Zhang Y, Ye K, Jun G, Hsi-Yang Fritz M *et al*: **An integrated map of structural variation in 2,504 human genomes**. *Nature* 2015, **526**(7571):75-81.
32. Bao W, Kojima KK, Kohany O: **Repbase Update, a database of repetitive elements in eukaryotic genomes**. *Mob DNA* 2015, **6**:11.
33. Oesper L, Satas G, Raphael BJ: **Quantifying tumor heterogeneity in whole-genome and whole-exome sequencing data**. *Bioinformatics* 2014, **30**(24):3532-3540.
34. Chin CS, Peluso P, Sedlazeck FJ, Nattestad M, Concepcion GT, Clum A, Dunn C, O'Malley R, Figueroa-Balderas R, Morales-Cruz A *et al*: **Phased diploid genome assembly with single-molecule real-time sequencing**. *Nat Methods* 2016, **13**(12):1050-1054.
35. Jain M, Koren S, Miga KH, Quick J, Rand AC, Sasani TA, Tyson JR, Beggs AD, Dilthey AT, Fiddes IT *et al*: **Nanopore sequencing and assembly of a human genome with ultra-long reads**. *Nat Biotechnol* 2018, **36**(4):338-345.
36. Zheng GX, Lau BT, Schnall-Levin M, Jarosz M, Bell JM, Hindson CM, Kyriazopoulou-Panagiotopoulou S, Masquelier DA, Merrill L, Terry JM *et al*: **Haplotyping germline and cancer genomes with high-throughput linked-read sequencing**. *Nat Biotechnol* 2016, **34**(3):303-311.
37. Xia LC, Bell JM, Wood-Bouwens C, Chen JJ, Zhang NR, Ji HP: **Identification of large rearrangements in cancer genomes with barcode linked reads**. *Nucleic Acids Res* 2018, **46**(4):e19.
38. Bell JM, Lau BT, Greer SU, Wood-Bouwens C, Xia LC, Connolly ID, Gephart MH, Ji HP: **Chromosome-scale mega-haplotypes enable digital karyotyping of cancer aneuploidy**. *Nucleic Acids Res* 2017.

39. Ono Y, Asai K, Hamada M: **PBSIM: PacBio reads simulator--toward accurate genome assembly**. *Bioinformatics* 2013, **29**(1):119-121.
40. Yang C, Chu J, Warren RL, Birol I: **NanoSim: nanopore sequence read simulator based on statistical characterization**. *GigaScience* 2017, **6**(4):1-6.
41. Li H, Durbin R: **Fast and accurate short read alignment with Burrows-Wheeler transform**. *Bioinformatics* 2009, **25**(14):1754-1760.
42. Li H: **Toward better understanding of artifacts in variant calling from high-coverage samples**. *Bioinformatics* 2014, **30**(20):2843-2851.
43. Bashir A, Bansal V, Bafna V: **Designing deep sequencing experiments: detecting structural variation and estimating transcript abundance**. *BMC Genomics* 2010, **11**:385.
44. Zagordi O, Daumer M, Beisel C, Beerenwinkel N: **Read length versus depth of coverage for viral quasispecies reconstruction**. *PLoS One* 2012, **7**(10):e47046.
45. Liao Y, Smyth GK, Shi W: **featureCounts: an efficient general purpose program for assigning sequence reads to genomic features**. *Bioinformatics* 2014, **30**(7):923-930.
46. **SVEngine: Allele Specific and Haplotype Aware Structural Variants Simulator**. Bitbucket repository. https://bitbucket.org/charade/svengine. Accessed 26 June 2018.
47 Xia LC, Ai D, Lee H, Andor N, Li C, Zhang NR, Ji HP. **Supporting data for "SVEngine: an efficient and versatile simulator of genome structural variations with features of cancer clonal evolution**" GigaScience Database 2018. http://dx.doi.org/100473

TABLES

Table 1. Available features of structural variant simulators.

Use Cases	SVEngine	RSVsim	SCNVsim	VarSim	BAMsurgeon
**Copy number events**: deletions, tandem duplications	✓	✓	✓	✓	✓
**Copy number neutral events**: inversions, insertions, translocations	✓	✓	✗	✓	✓
**Phylogenetic clonal structure**: cancer clonal evolution tree model	✓	✗	✓	✗	✗
**Foreign sequence insertion:** virus integration	✓	✗	✗	✗	✓
**Non-human genome:** variable haploid template and ploidy	✓	✓	✓	✗	✓
**Not requiring pre-existing alignment:** without BAM input	✓	✓	✓	✓	✗
**Generate simulated contig:** with FASTA output	✓	✓	✓	✓	✓
**Generate simulated reads:** with FASTQ output	✓	✗	✗	✓	✗
**Generate simulated alignment:** with BAM output	✓	✗	✗	✗	✓
**Locus-specific variant ploidy:** allelic imbalance	✓	✗	✗	✗	✗
**Locus-specific variant frequency:** variable somatic allele frequency	✓	✗	✗	✗	✓
**Exact breakpoint:** specifiable at base pair resolution	✓	✓	✗	✗	✓
**Multiple sequencing libraries:** multiple insert size, read length, etc.	✓	✗	✗	✗	✗

Table 2. Runtime performance comparison.

15000 events at 30x coverage	SVEngine (64 cores)	SVEngine (1 core)	*RSVSim*	*SCNVSim*	*VarSim*	*BAMSurgeon*
FASTA output	*<10 min*	*< 10 min*	*10 hr*	*2 hr*	*Not Available*	*Not Available*
FASTQ output	*<20 min*	*5 hr*	*External*	*External*	*6 hr*	*Not Available*
BAM output	*2hr*	*5 days*	*External*	*External*	*External*	*>10 days*

Not Available: output format is not available

External: output format is only available through additional external software, thus not tested

FIGURES

Figure 1. Inputs, outputs and execution components of SVEngine. The flow of data was marked by gray arrows. The input, SVEngine functioning and output data spaces were color shaded. (*) Note: new file formats VAR, META and PAR were introduced by SVEngine for specifying specific variants (VAR) or variants' meta-distribution (META) to be simulated, or for specifying parameters for sequencing library and run (PAR). Please see the online manual for detailed explanations.

Figure 2. Simulating cancer evolution. (A) An example cancer evolution tree. The conditional fraction in each internal node represents the fraction of cell population gaining the next structural variation, which is represented by the label of the internal node. (**B)** An example computation table to determine final variant frequency of each variation and cell population frequency of each terminal genotypes. **(C)** Integrated Genomics Viewer view of SVEngine simulated BAM data of five deletions following clonal structure in the example binary tree.

Figure 3. Expected read mapping features of structural variant prototypes. Rows – variant prototypes: 1) Deletion, 2) Insertion, 3) Duplication, 4) Inversion. Columns – mapping features: 1) Read coverage, 2) Read pair insert size, 3) Single end mapped read (HangingRead), 4) Soft clipped read or split mapped read (ClipSplitRead). X-axis is genomic coordinates. Y-axis is feature value/counts. Dashed orange line stands for expected feature value without alteration. Solid blue line stands for expected feature value with alteration. Dotted green bar denotes the breakpoint(s).

Figure 4. The core parallelized simulation algorithm of SVEngine. Here are two neighboring events SV_i and SV_j: a 50% deletion and a 100% deletion to be spiked-in. The

first deletion event spans blocks $B_{i_1}, B_{i_1+1}, \ldots, B_{i_n}$ and the second deletion event spans genome blocks $B_{j_1}, B_{j_1+1}, \ldots, B_{j_n}$. The genome blocks are shaded in blue while the ligation regions are shaded in orange. The resulting read pairs are represented by their coverage in black dash patterns. The parallel execution tasks were boxed in green color.

Figure 5. Simulation benchmark of SVEngine. We measured (**A**) True Positive Rate (TPR) and (**B**) False Discovery Rate (FDR) for the two SV callers (Lumpy and Manta) using SVEngine simulated WGS data with 10x, 25x, 50x, 75x and 100x coverage respectively. (**C**) We measured the concordance in R^2 between THetA2 estimated purity and ground truth with 10%, 25%, 50%, 75% and 90% tumor cell fractions, based on SVEngine simulated WGS data.

SUPPLMENTARY TABLES

Table S1 Simulation benchmark on NA12878. The table includes full data of NA12878 simulation results, including computed true positive rate (TPR) and false discovery rate (FDR) for overall performance when SV typing correctness was not enforced (‘all’) or for performance involving specific SV categories when SV typing correctness was enforced (‘del’, ‘ins’, ‘inv’, ‘dup’).

Table S2 Simulation benchmark on tumor heterogeneity. The table includes THetA2 estimated purity and input ground truth purity to SVEngine for the tumor heterogeneity simulation.

Figure 1. Inputs, outputs and execution components of SVEngine.

Click here to access/download;Figure;vs07_svengine_figure1.pdf

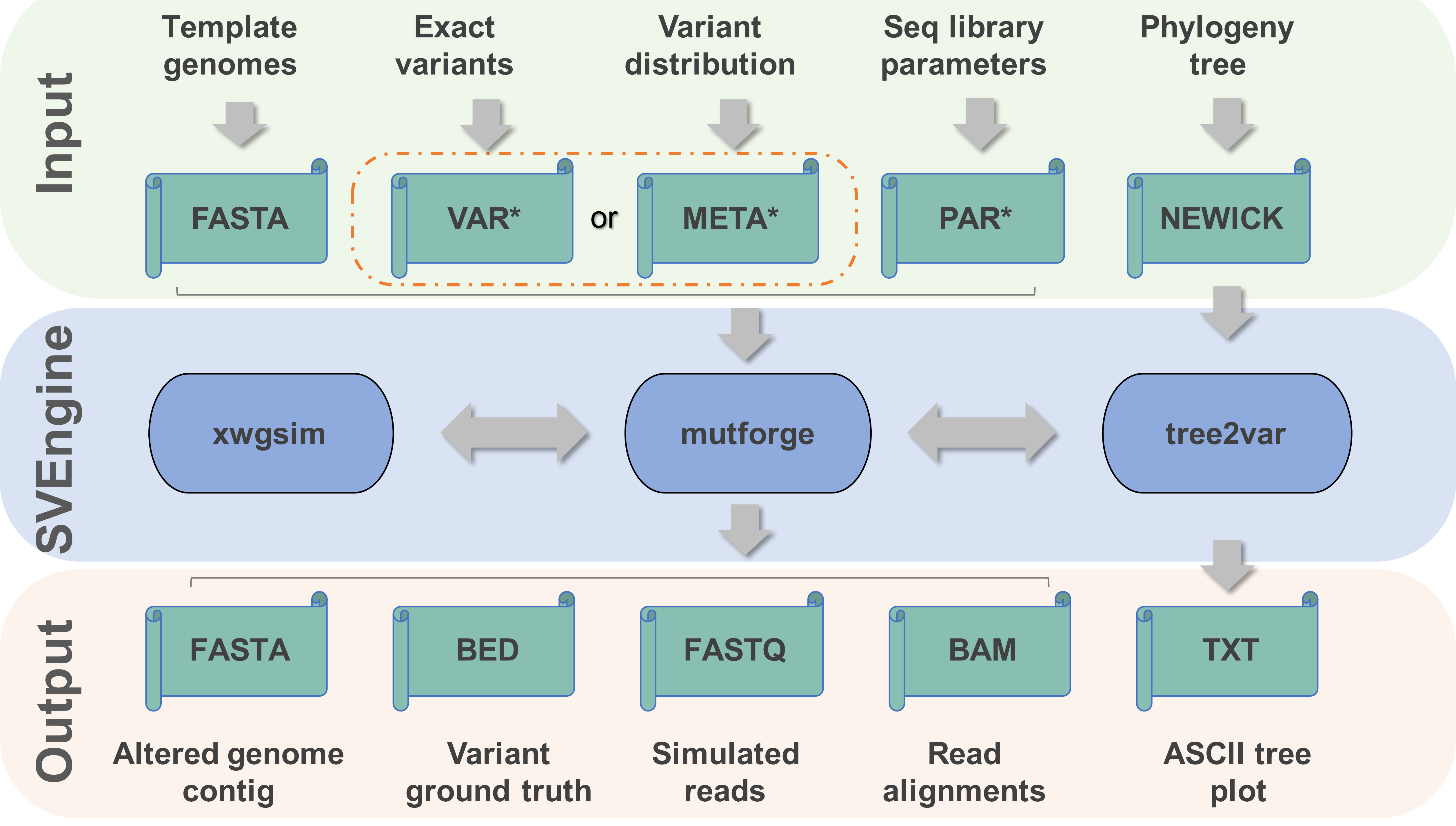

Click here to access/download;Figure;Xia_svengine_figure2.pdf

Figure 2. Simulating cancer evolution.

A An example binary tree clonal structure representation

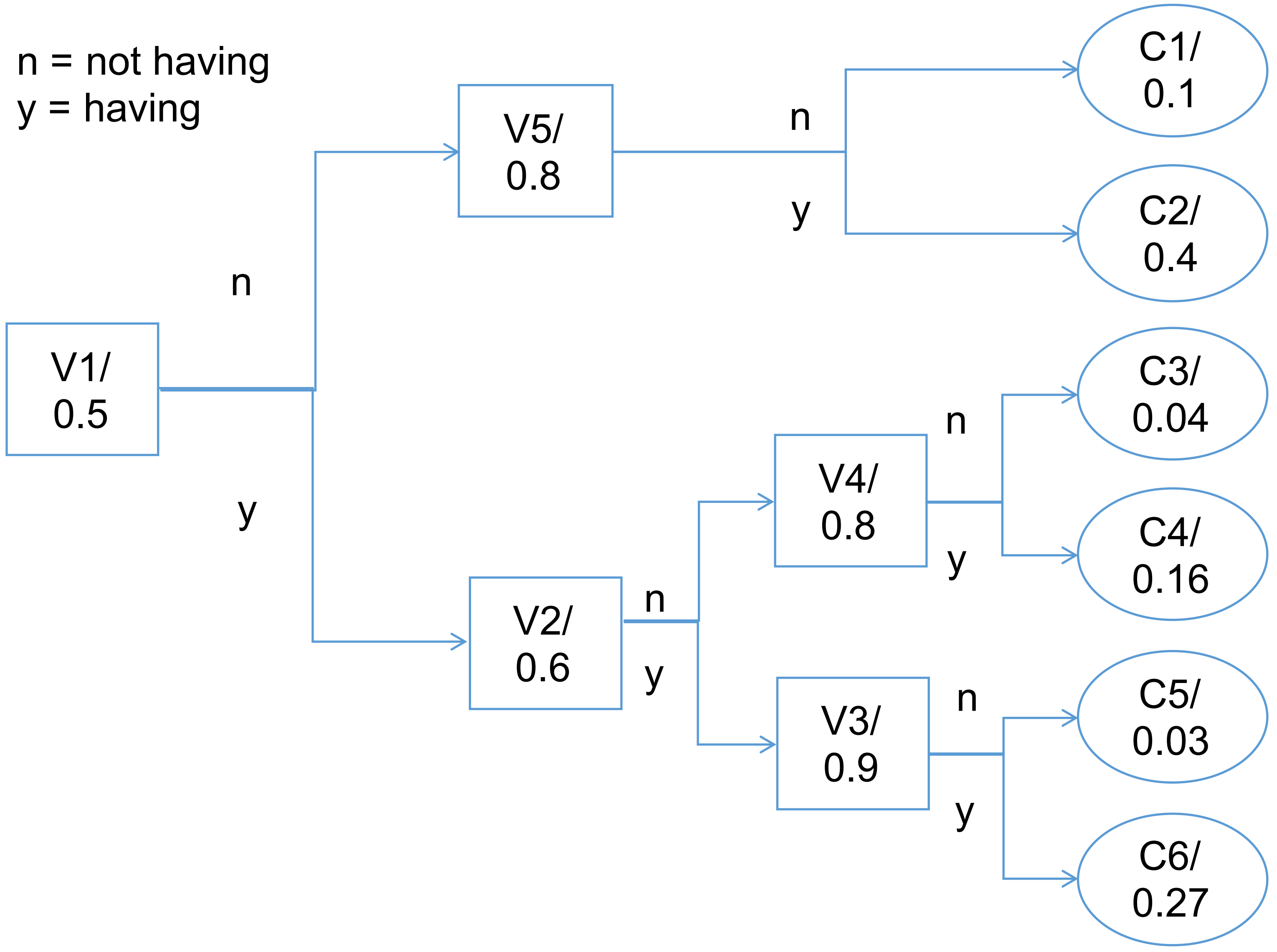

B Deriving cell subpopulations and variant frequencies

Cell Pop. Variants	Leaf Node	Internal Nodes / Variant Evolutionary Path					Cell Pop. Freq.
		V1	V2	V3	V4	V5	F(Ci)
Normal	C1	1	0	0	0	1	0.1
V5	C2	1	0	0	0	2	0.4
V1	C3	2	1	0	1	0	0.04
V1, V4	C4	2	1	0	2	0	0.16
V1, V2	C5	2	2	1	0	0	0.03
V1, V2, V3	C6	2	2	2	0	0	0.27
**Var. Cond. Fraction: f(Vi)**		0.5	0.6	0.9	0.8	0.8	
**Var. Total Fraction: F(Vi)**		**0.5**	**0.3**	**0.27**	**0.16**	**0.4**	

C Simulated BAM data of five deletions following clonal structure in the binary tree

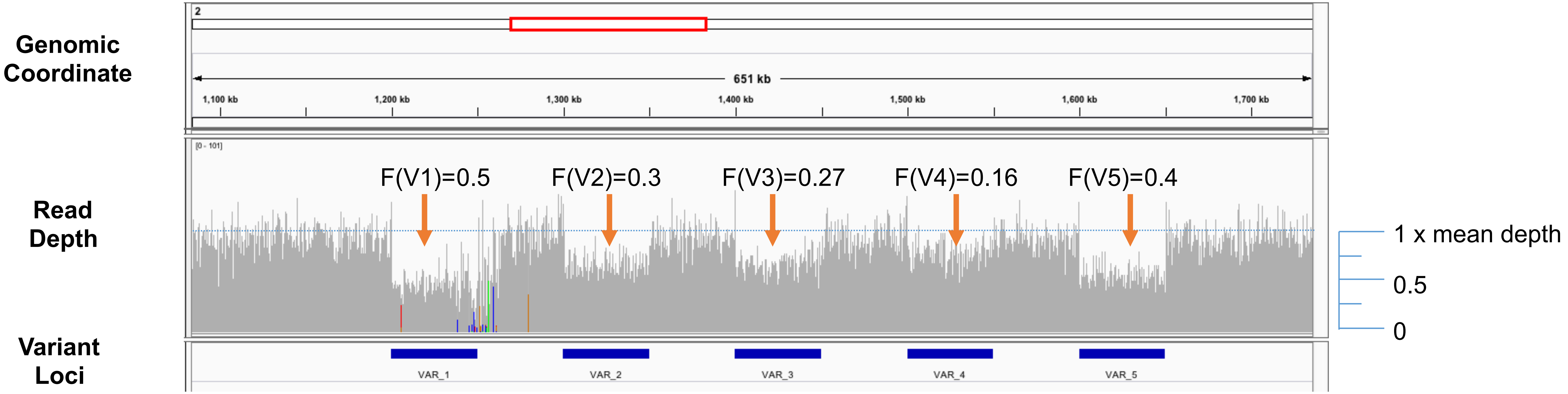

Figure 3. Expected read mapping features of structural variant prototypes.

Click here to access/download;Figure;Xia_svengine_figure3.pdf

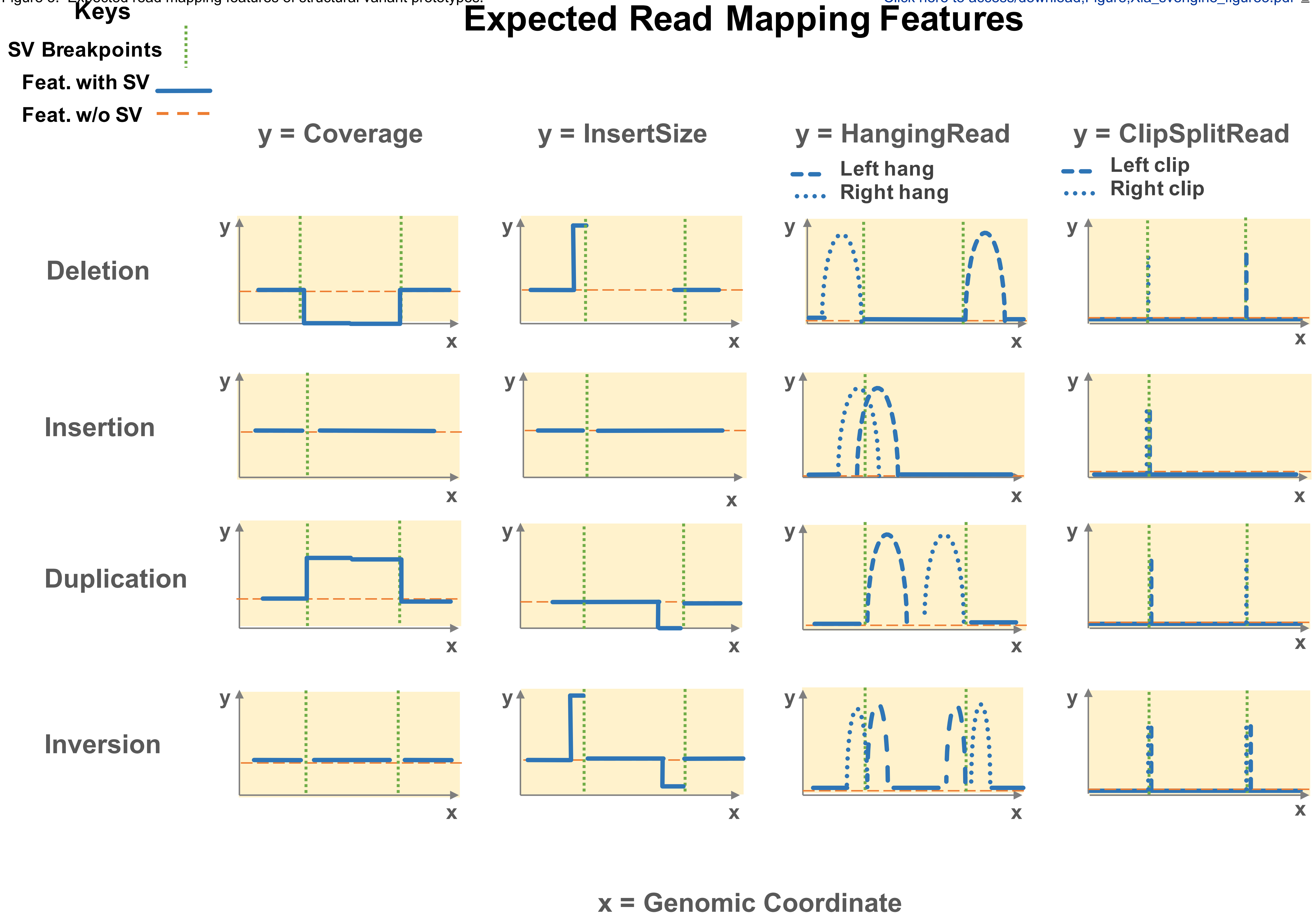

Figure 4. The core parallelized simulation algorithm of SVEngine.

Click here to access/download;Figure;Xia_svengine_figure4.pdf

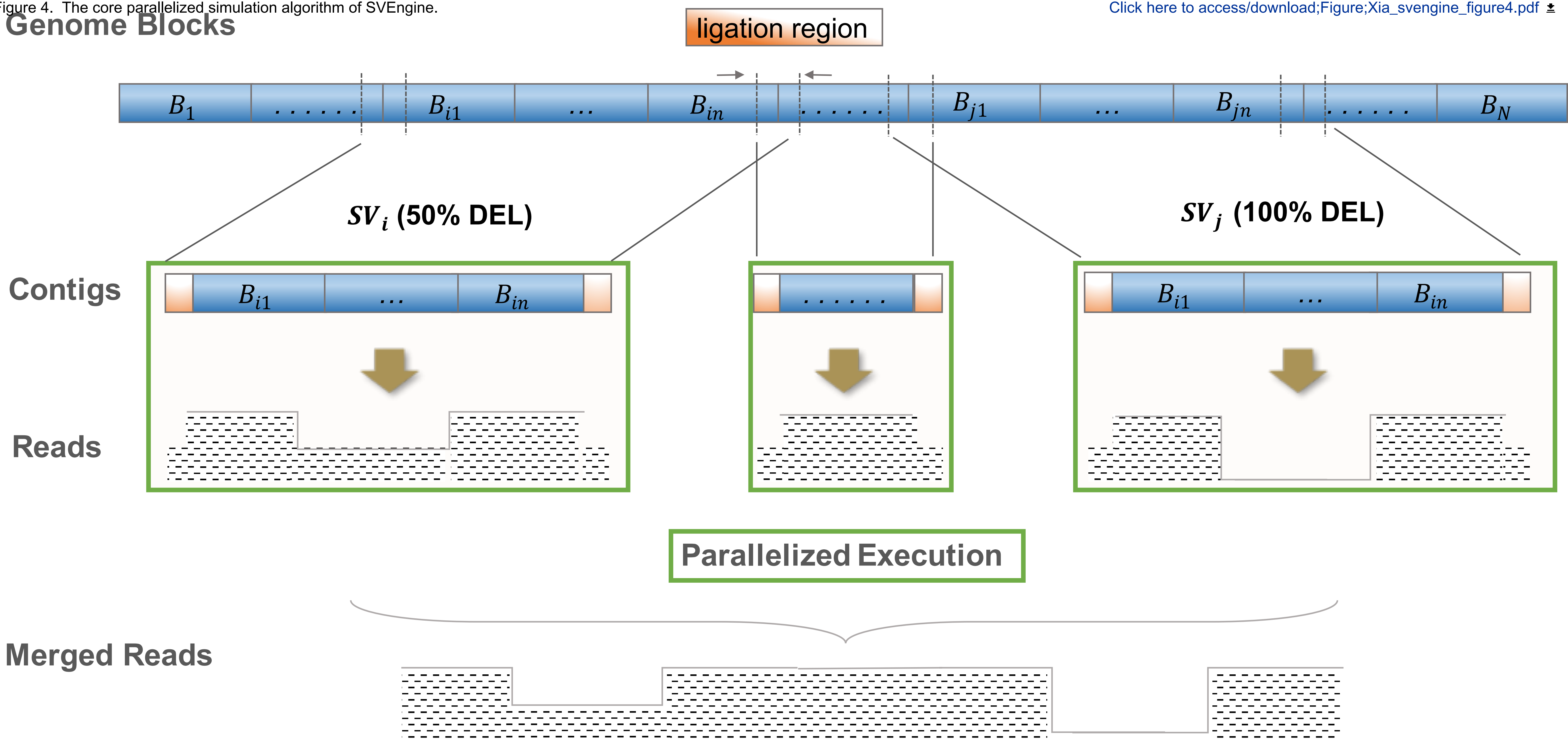

Figure 5. Simulation benchmark of SVEngine.

Click here to access/download;Figure;vs07_svengine_figure5.pdf

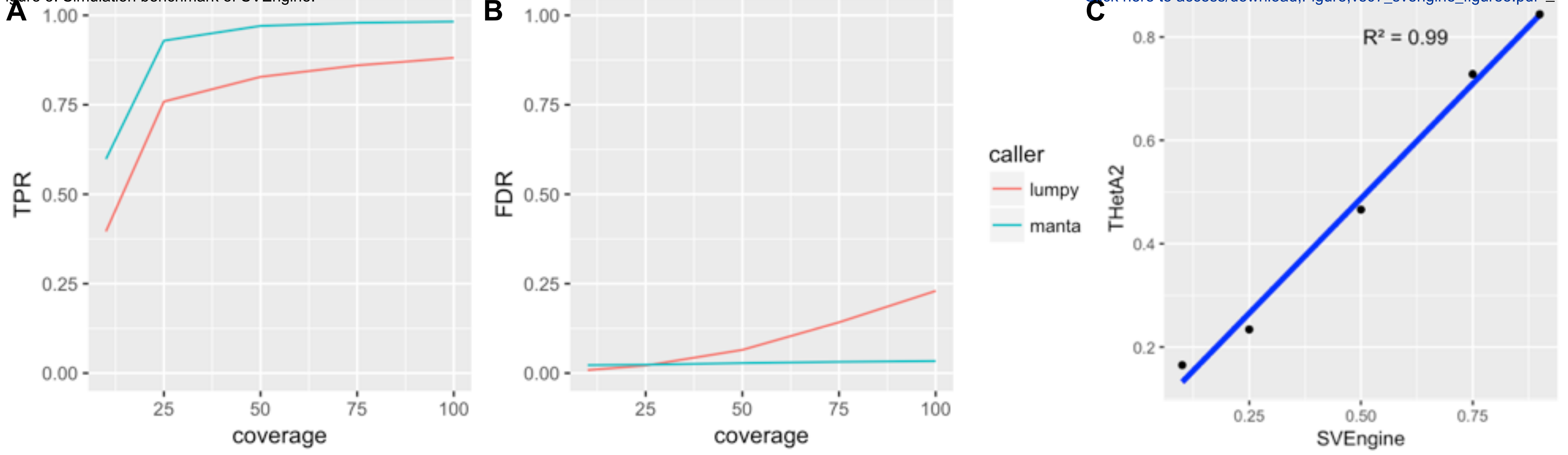

Supplementary Table

Click here to access/download
Supplementary Material
vs07_svengine_supp_tables.xlsx
