# Supplementary material for: SVEngine: an efficient and versatile simulator of genome structural variations with features of cancer clonal evolution
Source: Gigascience. 2018 Jul 5;7(7):giy081. doi: 10.1093/gigascience/giy081 (PMC6057526; doi:10.1093/gigascience/giy081)
Supplement: Response_to_Reviewer_Comments_Original_Submission.pdf [file giy081_response_to_reviewer_comments_original_submission.pdf]

Author's Response To Reviewer Comments

Close

We thank the editors and reviewers offered their great suggestions and comments based on the previous version of SVEngine manuscript. With this revision, we addressed all the issues raised by the reviewers and editors. And through the process, we had improved the overall quality of this manuscript. We hope the revised manuscript achieves the high standard of scientific publication of GigaScience. Thank you for your consideration.

Sincerely,

Hanlee Ji, MD

Associate Professor of Medicine
Stanford University School of Medicine

[Point-to-Point Response]

[Response to editors' comments]
Among the major points of the reviewers I'd like to highlight the need for benchmarking and evaluation of the simulations, to show that the result resembles real data (See the comments of reviewers 2 and 3).

Response:
We have performed additional benchmark simulation as suggested by the two reviewers. In the revision, we reported additional simulation findings and released the data to reproduce our results in the public domain (https://bitbucket.org/charade/svengine/wiki/Example). By applying two popular SV callers and one tumor heterogeneity estimator to these data and studying their performance, we further confirmed the validity of our simulations. For details, please see our point-to-point
responses to reviewer #3' specific comments.

A minor comment that came up during editorial discussion of the paper was that ample development is currently going into SV discovery / assembly software based on long read technology (PacBio / ONT), and we wondered whether it would be possible to amend the software in the future for this purpose? (this is a discretionary comment that you can decide to discuss in the paper, or not).
Response:
We thank the editors for raising this point to us and fully understand its importance. We added following discussion to the end of this manuscript as copied below:
"Structural variant analysis is a significant component of genomics research, which is continuously being improved by a growing set of available technologies, e.g. long read technologies such as the single-molecule, real-time (SMRT) sequencing by Pacific Biosciences [35] and the nanopore sequencing by Oxford Nanopore Technologies [36], or synthetic long read technologies (SLR) such as the Chromium droplet-based library preparations by 10X Genomics [37, 38]. As the empirical data from these technologies accumulate, platform specific read simulators like PBSIM [39] and NanoSim [40] will become increasingly available. Although the implementation will be non-trivial, the design of SVEngine is fully compatible with alternative read simulators. Going forward, we will

work with the community to expand SVEngine with more powerful features, such as multi-platform simulation and co-phased SNP simulation."
We noticed there are only a few currently published read simulators for Pacbio and ONT technologies such as PBSIM and NanoSim. The design of SVEngine would allow them to be integrated. It does require some familiarity of each read simulator's source code to do the proper source-code level modification. Since SVEngine is being publicized open source, we expect us, with the help from read simulator developers, will be able to implement these requested features in the near future.

- I believe you had discussed software licensing with one of my colleagues at submission if I read the notes on the file correctly. As discussed, please change the (non-OSI) Stanford license to an OSI-license such as, for example, BSD3 (https://opensource.org/licenses/BSD-3-Clause).
Response:
Yes. We had discussed the licensing options with the GigaScience editor's. The SVEngine software is now released as OSI compatible BSD3.

- Please register your new software application in the SciCrunch.org database to receive a RRID (Research Resource Identification Initiative ID) number and include this in your manuscript. This will facilitate tracking, reproducibility and re-use of your tool.
Response:
Yes. We have registered SVEngine with SciCrunch and the status is curated. The associated RRID is SCR_016235.

If you are able to fully address these points, we would encourage you to submit a revised manuscript to GigaScience.
Minor comment from editorial discussion: - The authors write: It is typically the normal germline cell as depicted here, or the first generation of cells bearing common somatic mutations that start becoming cancerous. In fact it is not typically the germline cell itself that becomes cancerous, but cells that carry a genome that (presumably) matches the genome of the germline.

Response:
We thank the editors for finding out this inaccuracy in our language. We have updated the manuscript to correctly convey the idea. The corrected sentence is:
"The root node represents the lowest common ancestor cells of all subpopulations of cancer cells. These are typically normal cells that carry a genome that matches a germline genome, subsequently from which somatic genetic alterations accrue as part of cancer development."

[Response to reviewer #1's comments]
Reviewer #1: This manuscript presents the design and implementation of a structural variant simulation software called SVEngine. Compared with related software, SVEngine provides new features, such as the simulation of locus-specific variant frequency. Parallelization optimization is also adopted to improve the computing speed of SVEngine. It is a topic of interest to the researchers in the related areas, but the paper needs minor reversion before acceptance for publication.
My comments are as follows: The author claimed the performance of SVEngine scales almost linearly with the added CPU power (1x, 15x and 48x times faster than the single-

process run) in the last paragraph of page five. The configuration of the computing server mentioned in the article (first paragraph of page six) is a computer equipped with one Intel i7-4790 CPU, which has four physical cores and supports eight threads by hyper-threading. If the threads don't do I/O and there's nothing else running, one thread per core will get you the best performance. However, that very likely not the case in the experiment (48x speedup on 4 physical core). As the computation performance is one of the highlights of SVEngine, the explanation of the acceleration should be provided in detail. Evaluation and analysis under different parallel scale are also needed, so as to locate the performance bottleneck and support the parallel speedup of the pipeline.

Response:
We thank the reviewer for the positive and constructive comments. To the reviewer's question, we apologize the CPU and computer server information was incorrect in the previous version, which caused the confusion. We can confirm that the performance was measured on a 4-CPU computer cluster, where each CPU has 16 physical cores. In total there is 64 cores that together give us a 48x speedup. The acceleration is almost linear and within our expectation. The corrected text is below:

"All run time were measured on a computer server with four Intel® Xeon E7-4850v4 CPUs (16 cores each CPU) and with 256 GB shared RAM."

To perform analysis under different parallel scales, we have provided additional benchmark data that supports SVEngine's efficient computational performance (see our newly added data for NA12878 and a cancer genome in the revised paper). In these experiments, we have successfully simulated ~ 20,000 in silico events in the entire human genome at 100X coverage without issues. To generate the final BAM file took about 1 day on a 32 physical cores (4 units of AMD Opteron 6386 SE, 8 physical cores each unit) and 256GB RAM server. We found the overall runtime was linearly bounded by the number of events being modeled and the total coverage and scaled nearly as a fraction of the available cores. We can identify the real step has the potential to be a bottleneck is to map the short reads toward human genome, which was actually performed with bwa. It is out the scope of SVEngine's design and is optionally substituted if necessary. We hope we can convince the reviewer, SVEngine is an efficient simulator, given that we have simulated dozens of human genomes with tens of thousands of all types of events and varied coverages without issues (these numbers are in realistic range for total SVs based on literature).

[Response to reviewer #2's comments]
Reviewer #2: SVEngine is a welcome addition to a niche of variant simulation tools that can produce structural rearrangements for the purpose of benchmarking SV detection tools. SVEngine provides a few features not found elsewhere, the most notable perhaps is the ability to simulate subclonality with a bifurcating tree model.
Given that this is a tool intended to be used for benchmarking, it would be helpful to see some benchmarking data in the manuscript to reassure the reader that SVEngine does indeed create SVs of each type supported that are detectable with standard SV calling tools.
Response:
We thank the reviewer for the suggestion. In the revision we have added substantial new benchmark data to further validate the performance of SVEngine using existing SV calling tools. Since the newly added benchmark was structured based on reviewer #3's first comment, we refer reviewer #2 to our response there. The first part of new benchmark data we described there addressed this comment.

What is the utility of supporting multiple insert sizes within the same simulation? How often, in recent practice, does one encounter a sequencing run that was constructed with multiple insert sizes?
Response:
Regarding the reviewer’s question on the utility of multiple insert size library, we agree that such use is not common in our experience. However, there are circumstances where this occurs. For example, we know of experiments where it was intentionally designed three libraries of different insert sizes in the hope to perform sensitive and comprehensive detection for deletions. Their rationale was, for each insert size, the effective detection range is at least two standardard deviations away from the mean. So, the use of multiple insert size would allow the combined effective detection range to cover the full size spectrum of deletions. However, since that design was not prospectively validated with bioinformatics tools using simulation data, it had caused significant challenge in downstream analysis. Now with SVEngine, the pro and cons of multi-insert design, if desired, can be studied before sequencing.
Another example involves mate-pair sequencing where larger inserts, 2 kb or more are used to improve the detection of structural variants. To have benchmark in silico data sets to reflect the properties of these libraries would require varying the insert size.
The multiple insert size simulation is also useful retrospectively, in particular when multi-modal or asymmetric insert-size distributions arise from real data. In principle, such multi-modal or asymmetric distributions can be approximated by a mixture of normal densities. Using SVEngine to generate similarly distributed data with known ground truth will enable one to benchmark the performance and robustness of intended bioinformatics analysis. Note, this task would not be possible with the real data only because the ground truth would not be available. We thank the reviewer for asking this great question and we have added our answer to our online FAQ.

I think the items in Fig 1 could use a bit more explanation. For example, from the figure, 'sequencing library' might be interpreted to mean an actual fastq file, but it actually means a file with information on paired end read distributions as per the example on bitbucket, and "PAR" is just an arbitrary extension.
Response:
We thank the reviewer for the valuable suggestions. We have updated Figure 1 to accommodate them accordingly. The newly introduced file formats like VAR, META and PAR are starred. We briefly explained them in the figure caption and pointed readers to the online manual for additional information. The added caption text is copied here:
“(*) Note: new file formats VAR, META and PAR were introduced by SVEngine for specifying specific variants (VAR) or variants’ meta-distribution (META) to be simulated, or for specifying parameters sequencing library and run (PAR). Please see the online manual for detailed explanations.”

Page 15, lines 51-53 "In addition, xwgsim adds a procedure to the popular NGS simulator wgsim [31], which rejects a new read pair at 50% chance if any of its two ends originates in a ligation region." -- I wasn't able to work out why this is necessary, could you clarify?
Response:
We apologize for the insufficient description. We defined the ligation region as a segment of

haploid sequence where two adjacent contigs to be simulated overlaps (see Figure 4). The ligation region is employed to ensure proper and continuous transition from simulating reads from the first contig to the second. That also means a ligation region would be simulated twice – once along with the first contig, and then along with the second contig. To compensate for potential double coverage in a ligation region, we implemented an adjusted read generation procedure in xwgsim, which only simulates 50% of the intended read coverage within the region for one contig. The modified text is copied here:

"Briefly, we define the ligation region as a segment of haploid sequence where two adjacent contigs being simulated overlaps. The ligation region is employed to ensure proper and continuous transition from simulating reads from the first contig to the second. That also means a ligation region would be simulated twice – once along with the first contig, and along with the second contig. To compensate for potential double coverage in a ligation region, we implemented an adjusted read generation procedure in xwgsim, which only simulates 50% of the intended read coverage within the region for one contig."

Is xwgsim integral to running SVEngine or could another read simulator be swapped in e.g. ART? I ask because wgsim is mainly aimed at Illumina data but simulators may exist for other data types and the ability to use them would extend the usefulness of SVEngine.

Response:

The short answer is yes. To make possible parallelized processing by SVEngine, in xwgsim, we modified the source code of wgsim to properly carry out contig-wise simulation employing ligation regions so as to precisely achieve desired simulation. xwgsim is thus integral to SVEngine.

Nonetheless, the source-code level modification as we did to wgsim is not complicated, which is likely also doable with other open source reader simulators and for other types of sequencing platforms, e.g., ART as mentioned here. It, however, does require some familiarity of the read simulator's source code. Since SVEngine is to be published open source, we expect, with help from read simulator developers, could implement such features, as the community getting actively engaged and coming up with these requests.

Similarly, it wasn't clear how configurable BAM generation was: suppose I want to use bowtie and not bwa or whatever aligner is the default - is this possible?

Response:

The short answer is yes. The direct BAM generation of SVEngine is currently tided to BWA MEM with default parameters. However, if that is not desired behavior, one can always take over after the FASTQ files have been generated and apply any available aligner as wishes. So, what has been asked is not only possible but easily doable by setting the option '-x fastq' to SVEngine. It is actually our current default to generate FASTQ files as end points of simulation (please refer to our online manual). The simulated read pairs will be saved into two FASTQ files 'xxx.fq1' and 'xxx.fq2', which are suitable input to bowtie or other aligners of choice. We have included this Q&A information in our online FAQ.

This is referring to the program itself and not the paper: Is there an intuitive explanation for what 'trunksize' and 'plansize' mean?

Response:

Yes. We intuitively explained these terms below and the explanations were added to the online FAQ and the online manual.

Trunksize is a term we used to define the size of parallelly processed haploid contigs when no structural variant is present (non-variant contigs). The divide of non-variant contigs into

trunks enables concurrent simulation of reads and their efficient merge with reads from variant contigs. The current default trunksize is 10 Megabase, which, if considering no variants to be simulated at all, SVEngine will distribute the overall simulation to around 300 trunks for the human genome. In practice, the parameter should be considered in conjunction with the number of processors available. Intuitively, if the number of processors is 30, and by the default trunksize 1MB, each processor will process around 10 trunks before finishing. In this case, the jobs are well distributed. But if the trunksize was chosen as 1 GB, then only around 3 trunks will be distributed parallelly and the rest 27 processors will be idling, which reduces SVEngine's efficiency and increases its running time, because it has to wait for the bigger sized (now 1GB) trunk simulation to finish. The rule of thumb is to choose trunksize as a fraction of what the genome size divided by the number of processors. The trunksize, however, does not affect the task distribution of variant contigs as these contigs are always created and distributed per variant basis.
Plansize is a term we used to describe the coarse grain unit size when we try to randomly distribute a desired number of structural variants genome-wide. It is needed when the input is a META file, which specifies a distribution of structural variants to be simulated. The current default is 100 Kilobase. Intuitively, for the human genome, the default is to divide it into around 300,000 units, all of which are marked available at the beginning. SVEngine then randomly samples from the available units to accommodate the next variant and mark the relevant units unavailable. The process continues until the desired distribution is achieved. For each variant, depending on its size and type, it could take a contiguous segment of one or more units. If the specified distribution turns out not accommodatable with current plansize, SVEngine will report an informative error and stop before any actual read simulation. One can then retry the simulation with reduced plansize or opt for a less ambitious simulation.

A few notes on the comparison with BAMSurgeon, the various points made are largely fair, but there are a few features the authors have missed. BAMSurgeon does support insertions including insertions of arbitrary sequences (e.g. viral sequences) through the INS type (see manual, pg 9-10). BAMSurgeon also does output the contigs generated before and after SV spike-in - they're in the addsv_logs_* directory after the run is complete. This isn't well-documented however. Finally, the user is able to specify per-variant allele fraction through the -c/--cnvfile option, although it is admittedly a bit arcane (page 4 of the manual has an explanation). These omissions are perhaps understandable to an extent but it raises the question of whether features have similarly been missed for the other tools compared to SVengine in this paper.
Response:
We thank the reviewers for pointing out the details regarding BAMSurgeon which we have overlooked. We acknowledge those valid points and have updated our comparison table to tick on the 'locus-specific variant frequency', 'foreign sequence insertion' and 'generate simulated contig' boxes for BAMSurgeon. We have rechecked publications and manuals of other tools and reaffirmed other comparisons made in the table.

[Response to reviewer #3's comments]
Reviewer #3: The authors present an SV simulation framework that is fast, easy to use, and the bitbucket documentation is good. Making heterogeneous SV datasets is very important to test new SV detection methods, and SVEngine makes this much easier.
I do have one major issue:
There needs to be an evaluation of the simulations to show that the result resembles real

data. For a "normal" case, this could be as easy as simulating NA12878's SVs, then running a handful of SV callers on both the real and simulated data and show that the results are similar. Given the title of the paper, the authors also need to show that the clonal populations that were specified are observed in the data. Something like THetA (L. Oesper Bioinformatics, 2014) or one of its successors could be used here.
Response:
We first thank the reviewer for the positive and constructive feedbacks. As suggested by the reviewer, we included two additional set of benchmark data with this revision.
In the first benchmark:
“We additionally validated SVEngine’s correctness by applying popular structural variant callers and a tumor heterogeneity estimating tool to SVEngine generated WGS data and benchmarked their performance with SVEngine’s input ground truth. In the first benchmark, we simulated WGS data for a well-studied individual NA12878 based on her known variants [31]. We applied commonly used SV callers: Lumpy [2] and Manta [10] and computed performance metrics such true positive rate (TPR or sensitivity) and false discovery rate (FDR) for the callers at different simulated coverage. In total, we simulated 20759 structural variants for NA12878 with exact genotype and breakpoint information. The set included 19034 deletions, 1150 duplications, 328 inversions and 247 insertions. These insertions included 91 LINE1, 58 ALU and 9 SVA mobile element insertions, for which we determined the exact inserted sequence using RepBase [32].”
In the second benchmark:
“To further validate SVEngine’s correctness in simulating various mutant allele frequency, we simulated a series of WGS data representing two-population mixtures of tumor and normal genomes at a chosen grade of tumor purities. We derived copy number segment files from the SVEngine simulated data set and ran the tumor heterogeneity caller THetA2 [33] to infer the mixing subpopulation frequency.”

The methods and results details of these benchmarks were added and were highlighted in the revision. These include a newly added Figure 5 and two newly added Supplementary tables S1 and S2. In summary, commonly used SV callers, Manta and Lumpy, generally performed well on calling and typing deletions, inversions and duplications with SVEngine simulated data. Calling and typing insertions is so far the most challenging task for SV analysis and the capability of SVEngine to simulate various insertions can aid the further development of insertion callers. Also, the copy number profiles derived from SVEngine simulated tumor and normal mixture WGS data are suitable input to standard tumor heterogeneity tools such as THetA2 for reliably estimating tumor purities, as demonstrated by good correlation and small residual errors between THetA2 estimates and input ground truth purities.
The results from both benchmarks have further validated the correctness and efficiency of SVEngine and the inclusion of which has strengthened the manuscript greatly.

I also have a suggestion:
This method would be much more powerful if it could simulate a diploid (or more) genome with accompanying SNPs properly phased. I would expect clonal evolution methods to use both SNPs and SVs, and that phasing these events would be quite powerful. If SVEngine supported phased simulations, then it would have a wider audience.
Response:
We thank the reviewer with this great suggestion and agreed such simulation would be more powerful. Simulating SNPs and small indels require additionally domain knowledge and a tool encompassing both requires extensive benchmark work that together would surpass the

scope of this manuscript. But we will definitely consider this suggestion in our future development of SVEngine.

Close
