# Supplementary material for: SVEngine: an efficient and versatile simulator of genome structural variations with features of cancer clonal evolution
Source: Gigascience. 2018 Jul 5;7(7):giy081. doi: 10.1093/gigascience/giy081 (PMC6057526; doi:10.1093/gigascience/giy081)
Supplement: Reviewer_1_Report_(Original_Submission) -- Shengjie Gao, Ph.D — 2/18/2018 Reviewed [file giy081_reviewer_1_report_(original_submission).pdf]

Reviewer Report

Title: **SVEngine: an efficient and versatile simulator of genome structural variations with features of cancer clonal evolution**

Version: **Original Submission** **Date:** 2/18/2018

Reviewer name: **Shengjie Gao, Ph.D**

Reviewer Comments to Author:

This manuscript presents the design and implementation of a structural variant simulation software called SVEngine. Compared with related softwares, SVEnigine provides new features, such as the simulation of locus-specific variant frequency. Parallelization optimization is also adopted to improve the computing speed of SVEngine.It is a topic of interest to the researchers in the related areas, but the paper needs minor reversion before acceptance for publication. My comments are as follows:The author claimed the performance of SVEngine scales almost linearly with the added CPU power (1x, 15x and 48x times faster than the single-process run) in the last paragraph of page five. The configuration of the computing server mentioned in the article (first paragraph of page six) is a computer equipped with one Intel i7-4790 CPU, which has four physical cores and supports eight threads by hyper-threading. If the threads don't do I/O and there's nothing else running, one thread per core will get you the best performance. However that very likely not the case in the experiment (48x speedup on 4 physical core). As the computation performance is one of the highlights of SVEngine, the explanation of the acceleration ration should be provided in detail. Evaluation and analysis under different parallel scale are also needed, so as to locate the performance bottleneck and support the parallel speedup of the pipeline.

Level of Interest

Please indicate how interesting you found the manuscript: An article whose findings are important to those with closely related research interests

Quality of Written English

Please indicate the quality of language in the manuscript: Needs some language corrections before being published

Declaration of Competing Interests

Please complete a declaration of competing interests, considering the following questions:

- Have you in the past five years received reimbursements, fees, funding, or salary from an organisation that may in any way gain or lose financially from the publication of this manuscript, either now or in the future?
- Do you hold any stocks or shares in an organisation that may in any way gain or lose financially from the publication of this manuscript, either now or in the future?
- Do you hold or are you currently applying for any patents relating to the content of the manuscript?

- Have you received reimbursements, fees, funding, or salary from an organization that holds or has applied for patents relating to the content of the manuscript?
- Do you have any other financial competing interests?
- Do you have any non-financial competing interests in relation to this paper?

If you can answer no to all of the above, write 'I declare that I have no competing interests' below. If your reply is yes to any, please give details below.

I declare that I have no competing interests.

I agree to the open peer review policy of the journal. I understand that my name will be included on my report to the authors and, if the manuscript is accepted for publication, my named report including any attachments I upload will be posted on the website along with the authors' responses. I agree for my report to be made available under an Open Access Creative Commons CC-BY license (http://creativecommons.org/licenses/by/4.0/). I understand that any comments which I do not wish to be included in my named report can be included as confidential comments to the editors, which will not be published.

I agree to the open peer review policy of the journal

To further support our reviewers, we have joined with Publons, where you can gain additional credit to further highlight your hard work (see: https://publons.com/journal/530/gigascience). On publication of this paper, your review will be automatically added to Publons, you can then choose whether or not to claim your Publons credit. I understand this statement. Yes
