# Supplementary material for: SVEngine: an efficient and versatile simulator of genome structural variations with features of cancer clonal evolution
Source: Gigascience. 2018 Jul 5;7(7):giy081. doi: 10.1093/gigascience/giy081 (PMC6057526; doi:10.1093/gigascience/giy081)
Supplement: Reviewer_2_Report_(Original_Submission) -- Adam Ewing — 2/26/2018 Reviewed [file giy081_reviewer_2_report_(original_submission).pdf]

Reviewer Report

Title: SVEngine: an efficient and versatile simulator of genome structural variations with features of cancer clonal evolution

Version: Original Submission **Date:** 2/26/2018

Reviewer name: Adam Ewing

Reviewer Comments to Author:

SVengine is a welcome addition to a niche of variant simulation tools that can produce structural rearrangements for the purpose of benchmarking SV detection tools. SVengine provides a few features not found elsewhere, the most notable perhaps is the ability to simulate subcloanality with a bifurcating tree model.Given that this is a tool intended to be used for benchmarking, it would be helpful to see some benchmarking data in the manuscript to reassure the reader that SVengine does indeed create SVs of each type supported that are detectable with standard SV calling tools.What is the utility of supporting multiple insert sizes within the same simulation? How often, in recent practice, does one encounter a sequencing run that was constructed with multiple insert sizes?I think the items in Fig 1 could use a bit more explanation. For example, from the figure, 'sequencing library' might be interpreted to mean an actual fastq file, but it actually means a file with information on paired end read distributions as per the example on bitbucket, and "PAR" is just an arbitrary extension.Page 15, lines 51-53 "In addition, xwgsim adds a procedure to the popular NGS simulator wgsim [31], which rejects a new read pair at 50% chance if any of its two ends originates in a ligation region." -- I wasn't able to work out why this is necessary, could you clarify?Is xwgsim integral to running SVengine or could another read simulator be swapped in e.g. ART? I ask because wgsim is mainly aimed at Illumina data but simulators may exist for other data types and the ability to use them would extend the usefulness of SVengine.Similarly, it wasn't clear how configurable BAM generation was: suppose I want to use bowtie and not bwa or whatever aligner is the default - is this possible?This is referring to the program itself and not the paper: Is there an intuitive explanation for what 'trunksize' and 'plansize' mean? A few notes on the comparison with BAMSurgeon, the various points made are largely fair, but there are a few features the authors have missed. BAMSurgeon does support insertions including insertions of arbitrary sequences (e.g. viral sequences) through the INS type (see manual, pg 9-10). BAMSurgeon also does output the contigs generated before and after SV spike-in - they're in the addsv_logs_* directory after the run is complete. This isn't well-documented however. Finally, the user is able to specify per-variant allele fraction through the -c/--cnvfile option, although it is admittedly a bit arcane (page 4 of the manual has an explanation). These omissions are perhaps understandable to an extent but it raises the question of whether features have similarly been missed for the other tools compared to SVengine in this paper.

Level of Interest

Please indicate how interesting you found the manuscript: An article of importance in its field

Quality of Written English

Please indicate the quality of language in the manuscript: Needs some language corrections before being published

Declaration of Competing Interests

Please complete a declaration of competing interests, considering the following questions:

- Have you in the past five years received reimbursements, fees, funding, or salary from an organisation that may in any way gain or lose financially from the publication of this manuscript, either now or in the future?
- Do you hold any stocks or shares in an organisation that may in any way gain or lose financially from the publication of this manuscript, either now or in the future?
- Do you hold or are you currently applying for any patents relating to the content of the manuscript?
- Have you received reimbursements, fees, funding, or salary from an organization that holds or has applied for patents relating to the content of the manuscript?
- Do you have any other financial competing interests?
- Do you have any non-financial competing interests in relation to this paper?

If you can answer no to all of the above, write 'I declare that I have no competing interests' below. If your reply is yes to any, please give details below.

I declare that I have no competing interests

I agree to the open peer review policy of the journal. I understand that my name will be included on my report to the authors and, if the manuscript is accepted for publication, my named report including any attachments I upload will be posted on the website along with the authors' responses. I agree for my report to be made available under an Open Access Creative Commons CC-BY license (http://creativecommons.org/licenses/by/4.0/). I understand that any comments which I do not wish to be included in my named report can be included as confidential comments to the editors, which will not be published.

I agree to the open peer review policy of the journal

To further support our reviewers, we have joined with Publons, where you can gain additional credit to further highlight your hard work (see: https://publons.com/journal/530/gigascience). On publication of this paper, your review will be automatically added to Publons, you can then choose whether or not to claim your Publons credit. I understand this statement. Yes
